# Direct Neuronal Reprogramming: Bridging the Gap Between Basic Science and Clinical Application

**DOI:** 10.3389/fcell.2021.681087

**Published:** 2021-07-05

**Authors:** Lakshmy Vasan, Eunjee Park, Luke Ajay David, Taylor Fleming, Carol Schuurmans

**Affiliations:** ^1^Sunnybrook Research Institute, Biological Sciences Platform, Toronto, ON, Canada; ^2^Department of Laboratory Medicine and Pathobiology, University of Toronto, Toronto, ON, Canada; ^3^Department of Biochemistry, University of Toronto, Toronto, ON, Canada

**Keywords:** direct neuronal reprogramming, lineage conversion, transcription factors, micro-RNA, astrocytes, fibroblasts, small molecules, epigenetics

## Abstract

Direct neuronal reprogramming is an innovative new technology that involves the conversion of somatic cells to induced neurons (iNs) without passing through a pluripotent state. The capacity to make new neurons in the brain, which previously was not achievable, has created great excitement in the field as it has opened the door for the potential treatment of incurable neurodegenerative diseases and brain injuries such as stroke. These neurological disorders are associated with frank neuronal loss, and as new neurons are not made in most of the adult brain, treatment options are limited. Developmental biologists have paved the way for the field of direct neuronal reprogramming by identifying both intrinsic cues, primarily transcription factors (TFs) and miRNAs, and extrinsic cues, including growth factors and other signaling molecules, that induce neurogenesis and specify neuronal subtype identities in the embryonic brain. The striking observation that postmitotic, terminally differentiated somatic cells can be converted to iNs by mis-expression of TFs or miRNAs involved in neural lineage development, and/or by exposure to growth factors or small molecule cocktails that recapitulate the signaling environment of the developing brain, has opened the door to the rapid expansion of new neuronal reprogramming methodologies. Furthermore, the more recent applications of neuronal lineage conversion strategies that target resident glial cells *in situ* has expanded the clinical potential of direct neuronal reprogramming techniques. Herein, we present an overview of the history, accomplishments, and therapeutic potential of direct neuronal reprogramming as revealed over the last two decades.

## Introduction

Brain injury and neurodegenerative diseases are among the leading causes of disability-adjusted life years (DALYs) and deaths globally (GBD 2016 Neurology Collaborators, 2019). While etiologies differ, these neurological conditions share the common feature of neuronal loss in the brain and/or spinal cord, the major constituents of the central nervous system (CNS). Stroke, the most common type of brain injury results in lifelong cognitive and motor deficits, and can rob an individual of their capacity to participate productively in society and ultimately lead to early death (GBD 2016 Neurology Collaborators, 2019). Similarly, neurodegenerative disorders that impact the CNS are ultimately fatal and include Alzheimer’s disease (AD), frontotemporal dementia (FTD), other dementias (Korsnes and Winkler, 2020), Amyotrophic Lateral Sclerosis (ALS) (Xu et al., 2020), and Parkinson’s disease (PD) (GBD 2016 Parkinson’s Disease Collaborators, 2018). In all of these disorders, neuronal loss results in permanent damage as neurogenesis does not occur in most of the adult mammalian CNS. Indeed, there are only a few active neurogenic zones in the adult mammalian brain, including the ventricular-subventricular zone (V-SVZ) lining the lateral ventricles, which replenishes neurons in the olfactory bulb in mouse and the striatum in humans, and the subgranular zone (SGZ), which generates new neurons for the dentate gyrus in all mammals (Spalding et al., 2013; Ernst et al., 2014; Urban and Guillemot, 2014; Gotz et al., 2016; Ruddy and Morshead, 2017; Boldrini et al., 2018; Sorrells et al., 2018). The purpose of this review is to summarize the use of direct neuronal reprogramming strategies to convert somatic cells to induced neurons (iNs) as a novel method to create new neurons for brain repair, disease modeling and drug screening.

**FIGURE 1:**
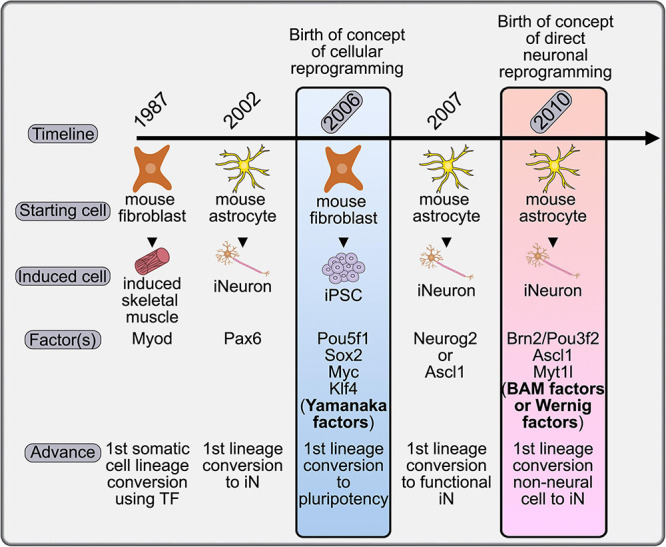
Timeline placing the concept of direct neuronal reprogramming in a historical context. The first evidence for somatic cell conversion was achieved in 1987 with the conversion of mouse fibroblasts to skeletal muscle cells. This procedure was adapted to the generation of iNeurons in 2002 and 2007 using astrocytes as a starting population, although not yet termed direct neuronal reprogramming. It was in 2006 that the field of cellular reprogramming was born with the demonstration that the Yamanaka factors could generate iPSCs. The formal launch of the direct neuronal reprogramming field came in 2010 with the identification of the BAM factors, which could convert fibroblasts, a non-neural cell type, to iNs without passing through a transient iPSC state.

The concept of cellular reprogramming was first formalized in 2006, with the Nobel Prize winning discovery that terminally differentiated murine (and later human) fibroblasts could be converted to a pluripotent state by the expression of a cocktail of four transcription factors (TFs): *Oct-3/4* (official gene name, *Pou5f1)*, *Sox2*, *Myc*, and *Klf4*, now colloquially called the Yamanaka factors (Takahashi and Yamanaka, 2006; Takahashi et al., 2007; Figure 1). The ability to differentiate iPSCs into a variety of somatic cell types has facilitated disease modeling, drug discovery, and led to pre-clinical and clinical trials that investigate the use of iPSC cell products as therapeutic treatments (reviewed in Jha et al., 2021; Qian and Tcw, 2021). The principal advantage to iPSC-derived cell products is that they lend themselves to a personalized medicine approach, as a patient’s own cells are used as donor cells, so the non-renewability of most cell types does not act as a limitation. However, there are justified concerns with the use of iPSC-derived neurons, including the potential association of pluripotency and tumorigenicity (Lee et al., 2013).

Direct cellular reprogramming involves the conversion of one heterologous cell type to another without passing through a pluripotent stage, and is considered a major advance for therapeutic innovation because it removes the tumorigenic potential of iPSC-derived cells (Lee et al., 2013). Although not formally defined as “cellular reprogramming,” the transdifferentiation of one somatic cell to another preceded the Yamanaka findings by several years with the demonstration that murine C3H10T1/2 embryonic fibroblast cells could be converted to a skeletal muscle identity by the misexpression of a single TF, the basic-helix-loop-helix (bHLH) gene, *Myod* (Davis et al., 1987; Figure 1). In this review we summarize efforts to reprogram a variety of somatic cells to iNs using combinations of TFs, miRNAs, and small molecules, and the application of such an approach to disease modeling, drug screening, and brain repair.

## Direct Neuronal Reprogramming *in vitro*

**FIGURE 2:**
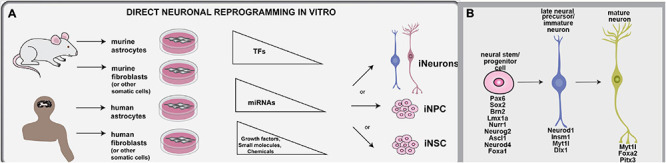
Methodologies used for direct neuronal reprogramming *in vitro.* **(A)** Direct neuronal reprogramming involves the direct conversion of somatic cells, usually astrocytes or fibroblasts, either directly into iNs or into iNSCs or iNPCs, which in turn can be differentiated into neurons. **(B)** Neuronal lineage conversion has been studies in murine and human cells for the most part, and involves the forced expression of lineage specifying transcription factors or miRNAs and the exposure to growth factors and other small molecules and chemicals that can make a pro-neurogenic environment.

The conversion of somatic cells to iNs has been achieved using both human and murine cells, primarily including astrocytes and fibroblasts, but also other starting cell populations. The TFs, miRNAs, and extrinsic cues used for direct neuronal programming *in vitro* are summarized herein (Figure 2A). Developmental neuroscience has paved the way for these studies, with most reprogramming cocktails incorporating TFs that drive the sequential conversion of neural stem cells (NSCs) to neural progenitor cells (NPCs), to immature and then mature neurons (summarized in Table 1; Figure 2B). Future studies will be required to determine how the molecular regulators used in these cocktails interact with each other to form gene regulatory networks (GRNs), and importantly, to compare and contrast the GRNs associated with neuronal reprogramming vs. those driving neurogenesis in the embryonic brain (e.g., Han et al., 2020).

**TABLE 1:** *In vitro* reprogramming.

**Source cell**	**Factors**	**Subtype**	**Efficiency**	**Functional outcome**	**References**
Human astrocytes	*Ascl1*, *Nurr1*, *Lmx1a*	DA	18.2 ± 1.5%	Dopamine release, electrophysiologically active, generate action potentials	Addis et al., 2011
Human fibroblast	*Brn2*, *Ascl1*, *Myt1l* (BAM)	Glutamatergic and GABAergic	16 ± 4.3% (embryonic) 4.3 ± 1.1% (postnatal)	Electrophysiologically active, generate action potentials	Pfisterer et al., 2011a
	BAM, *Neurod1*	Glutamatergic and GABAergic	2–4%	Electrophysiologically active, generate action potentials, form synapses	Pang et al., 2011
	BAM	Glutamatergic and GABAergic	19.5–20%	Electrophysiologically active, generate action potentials, form synapses	Vierbuchen et al., 2010; Chanda et al., 2014; Treutlein et al., 2016
	BAM, *Lmx1a*, *Foxa2*	Dopaminergic (DA)	10%	Electrophysiologically active, generate action potentials, dopamine synthesis	Pfisterer et al., 2011a
	*Ascl1*, *Nurr1*, *Lmx1a*	DA	6–10%	Electrophysiologically active, generate action potentials	Caiazzo et al., 2011
	*Ascl1*, *Neurog2*, *Nurr1*, *Pitx3*, *Sox2*	DA	1–2%	Electrophysiologically active, generate action potentials, Transplanted to striatum in PD rat model.	Liu et al., 2011
	*NEUROG2*, *SOX11*, *ISL1*, and *LHX3*	Cholinergic motor neurons	86–96%	Electrophysiologically active, generate action potentials	Liu et al., 2016
	*Brn2*, *Myt1l*, *miR-124*	Glutamatergic and GABAergic	4–8%	Electrophysiologically active, generate action potentials, form synapses	Ambasudhan et al., 2011
	*miR-124*, miR-9/9*with Neurod2/Ascl1 and Myt1l	Glutamatergic and GABAergic	80%	Electrophysiologically active, generate action potentials	Yoo et al., 2011
	*miR-124*, miR-9/9* with CTIP2, *DLX1*, *DLX2*, *and MYT1L*	Medium spiny neurons	76–93%, 80%	Electrophysiologically active, generate action potentials	Victor et al., 2014; Abernathy et al., 2017
Human vascular pericytes	*BAM*, *Tlx3 and miR-124*	Cholinergic neurons	80%	Not tested	Liang X. G. et al., 2018
Human cord blood cells	*Sox2*, *Myc*	Glutamatergic and GABAergic	80%	Electrophysiologically active, generate action potentials	Castano et al., 2014
Human pericytes	*Sox2*, *Ascl1*	Glutamatergic and GABAergic	48%	Electrophysiologically active, generate action potentials, form synapses	Karow et al., 2012
Human umbilical cord mesenchymal stem cells	*Sox2*, *Ascl1/Sox2*, *Neurog2*	Glutamatergic and GABAergic	50%	Electrophysiologically active, generate action potentials	Araujo et al., 2018
Mouse astrocytes	*Neurog2/Ascl1*	Glutamatergic and GABAergic	85%	Electrophysiologically active, generate action potentials, limited synapse formation	Berninger et al., 2007a
	*Neurog2*	Glutamatergic	70%	Electrophysiologically active, generate action potentials	McKay et al., 2010; Heinrich et al., 2011
	*Ascl1* + *Dlx1*	GABAergic	70%	Electrophysiologically active, generate action potentials	McKay et al., 2010; Heinrich et al., 2011
	*Neurog2*	Glutamatergic	54–73%	Electrophysiologically active, generate action potentials, transplantation to SVZ	Chouchane et al., 2017
	*Neurod4* + *Insm1*	Glutamatergic and GABAergic	40%; 60%	Electrophysiologically active, generate action potentials	Masserdotti et al., 2015; Rao et al., 2021
	*Ascl1/Neurog2 with Bcl2*	Glutamatergic and GABAergic	60–80%	Electrophysiologically active, generate action potentials	Gascon et al., 2016
Mouse cerebellar astrocytes	*Neurog2/Ascl1*	GABAergic neurons	54–73%	Electrophysiologically active, generate action potentials, engrafted in SVZ, and migrated to olfactory bulb	Chouchane et al., 2017
Astrocytes and fibroblasts	*Ascl1*, *Phox2b*, *AP2a*, *Gata3*, *Hand2*, *Nurr1*, *Phox2a*	Nor-adrenergic neurons	41.8%	Electrophysiologically active, generate action potentials	Li et al., 2019
Mouse fibroblasts	BAM	Glutamatergic and GABAergic	20%	Electrophysiologically active, generate action potentials	Vierbuchen et al., 2010
Mouse adult fibroblasts	BAM, *Lhx3*, *Neurog2/Hb9*, *Inl1*	Motor neurons	80.6%	Electrophysiologically active, generate action potentials, engrafted in chick neural tube	Son et al., 2011
MEF, adult tail tip fibroblasts (TTF)	*Ascl1*, *Nurr1*, *Lmx1a* (ANL)	DA	18.2 ± 1.5%	Electrophysiologically active, generate action potentials, dopamine release	Addis et al., 2011; Caiazzo et al., 2011
Mouse TTF	ANL, *Pitx3*, *Foxa2 En1*	DA	5–9%	Electrophysiologically active, generate action potentials, transplantation in striatum	Kim et al., 2011b
MEF	*Foxg1*, *Sox2*, *Ascl1*, *Dlx5*, *Lhx6*	GABAergic	9.4%	Electrophysiologically active, generate action potentials, transplanted to hippocampus	Colasante et al., 2015
Mouse adipocyte progenitor cells and hepatocytes	BAM	Glutamatergic and GABAergic	3–6%	Electrophysiologically active, generate action potentials	Yang et al., 2013
Mouse IPSC derived cardiomyocytes	BAM, *Neurod1*	Glutamatergic and GABAergic	35.5%	Electrophysiologically active, generate action potentials	Chuang et al., 2017
Mouse olfactory ensheathing cells	*Neurog2*	Glutamatergic	80%	Electrophysiologically active, generate action potentials, form synapses	Sun et al., 2019
Mouse-cochlear non-sensory epithelial cells, Spiral ganglion non-neuronal cells	*Ascl1*, *Neurod1*	Primary auditory neurons	49–55%	iNs extend projections	Noda et al., 2018
Mouse microglia	*Neurod1*	Glutamatergic and GABAergic	25–35%	Electrophysiologically active, generate action potentials	Matsuda et al., 2019
Rat fibroblast	ANL	DA	7%	90% of the DA transplanted into rat model.	Dell’Anno et al., 2014
Rat fibroblast	BAM	iN	0.4–5.9%	Transplant to striatum or hippocampus	Torper et al., 2013
***In vivo* cell reprogramming**					
Proliferating Glial cells	*Neurog2*, FGF, EGF	Glutamatergic	72 ± 5%	Not tested	Grande et al., 2013
Astrocytes	*Neurog2*, DAPT	Glutamatergic and GABAergic	50% (cortex), 30% (cerebellum), 20% (SC)	Not tested	Hu et al., 2019
Astrocytes	*Neurog2*, *Nurr1*	Glutamatergic and GABAergic	80%	Electrophysiologically active, generate action potentials, form synapses, long distance axonal projections	Mattugini et al., 2019
Striatal astrocytes	*Ascl1*, *Neurod1*, *Nurr1*	DA	16%	Electrophysiologically active, generate action potentials, motor behavioral recovery	Rivetti di Val Cervo et al., 2017
Striatal Neurons	*Sox2*, *Nurr1*, *Lmx1a and Foxa1* TFs and Valproic acid,	DA	79%	Electrophysiologically active, generate action potentials, dopamine restoration	Niu et al., 2018
Cortical astrocytes	*Neurod1*	Glutamatergic neurons	92.8%	Electrophysiologically active, generate action potentials	Guo Z. et al., 2014
NG2 glia	*Neurod1*	Glutamatergic and GABAergic	42.5%	Electrophysiologically active, generate action potentials	Guo Z. et al., 2014
Striatal microglia	*Neurod1*	Glutamatergic and GABAergic	25–35%	Electrophysiologically active, generate action potentials, and spontaneous synaptic currents	Matsuda et al., 2019
Reactive astrocytes	*Neurod1*	Glutamatergic and GABAergic	70%	Electrophysiologically active, generate action potentials, motor behavior improvement	Chen et al., 2020
Striatal non-reactive astrocytes	*Neurod1*	Glutamatergic neurons	80%	Not tested	Brulet et al., 2017
Astrocytes	*Neurod1*	Glutamatergic	20–27%	Improved locomotor activities and functional recovery	Livingston et al., 2020
Striatal astrocytes	*Neurod1*, *Dlx2*	GABAergic medium spiny neurons	80%	Electrophysiologically active, generate action potentials, extension of life span, and improved motor functions	Wu et al., 2020
					

### *In vitro* Reprogramming of Astrocytes to iNs

#### Transcription Factor-Based Reprogramming of Astrocytes to iNs

The first evidence for direct neuronal reprogramming came in 2002, with the conversion of murine postnatal day (P) 5-11 astrocytes to iNs by the forced expression of *Pax6*, a homeodomain TF with an essential role in cortical development (Heins et al., 2002; Figure 1). *Pax6* repressed expression of astrocytic glial fibrillary acidic protein (GFAP) and initiated expression of the neuronal marker, β-tubulin-III (Heins et al., 2002). Even though the term “direct neuronal reprogramming” was not used in the Heins et al. (2002) study, and was only later coined in 2010 (Vierbuchen et al., 2010), Heins et al. (2002) were the first to demonstrate that a terminally differentiated somatic cell fate could be converted to a neuron, and these “reprogrammed” cells can thus be referred to as iNs.

In 2007, iNs were generated from murine P5-7 cortical astrocytes by expressing one of two proneural bHLH genes: *Neurogenin 2* (*Neurog2*) or *Ascl1* (previous gene name, *Mash1*), and were shown to be functional as they could fire action potentials (Berninger et al., 2007a). In the embryonic forebrain, *Neurog2* and *Ascl1* promote neurogenesis and specify neuronal subtype identities: *Neurog2* specifies an excitatory, glutamatergic neuronal phenotype and *Ascl1* specifies an inhibitory, GABAergic neuronal identity (reviewed in Oproescu et al., 2021). *Neurog2* and *Ascl1* also specify distinct glutamatergic and GABAergic neuronal fates, respectively, when they are overexpressed in multipotent NSCs from the adult mouse brain (Berninger et al., 2007b), although this is an example of induced differentiation and not trans-differentiation, the focus of this review.

When P5-P7 murine astrocytes were lineage converted to iNs, *Neurog2*-iNs expressed Tbr1, a T-box TF that is a marker of maturing glutamatergic neurons. However, *Ascl1*-iNs did not express markers of a GABAergic fate (Berninger et al., 2007a). Moreover, even though both *Neurog2-* and *Ascl1*-iNs fired action potentials, synapse formation was limited (Berninger et al., 2007a). These limitations were overcome by changing the mode of gene delivery to a retroviral construct that was less prone to silencing and by using a stronger CAG promoter that achieved higher, sustained levels of proneural gene expression (Heinrich et al., 2010, 2011). With these modifications, *Neurog2*-iNs derived from postnatal astrocytes were glutamatergic, fired action potentials and formed synapses (Heinrich et al., 2010, 2011). In addition, when *Ascl1* was co-expressed with *Dlx2*, a homeodomain TF involved in specifying a GABAergic identity (reviewed in Wonders and Anderson, 2006), synaptically mature and electrically active iNs were generated (Heinrich et al., 2010, 2011). Interestingly, there are also regional differences in the response of astrocytic populations. For example, when *Neurog2* or *Ascl1* were expressed in early postnatal murine astrocytes isolated from the cerebellum, they generated inhibitory GABAergic iNs, which migrated to the olfactory bulb after transplantation into the subventricular zone (Chouchane et al., 2017). Conversely, only *Neurog2*, and not *Ascl1*, could transdifferentiate cortical astrocytes into excitatory glutamatergic neurons that integrated into the cortex (Chouchane et al., 2017). Thus, not only is it important to consider which TF to overexpress for lineage conversion, but also the starting somatic cell source, including its regional identity, as well as the mode of gene delivery and promoter choice.

To understand how *Neurog2* and *Ascl1* promote astrocyte-to-neuron lineage conversion, transcriptomic studies were performed after the expression of either factor in P6-P7 murine astrocytes (Masserdotti et al., 2015). While both proneural TFs rapidly induce neuronal differentiation and alter gene expression, there is only a ∼3% overlap in induced genes, consistent with the observation that these TFs specify distinct neuronal phenotypes in the embryo (Masserdotti et al., 2015). One of the common genes activated by both *Neurog2* and *Ascl1* is *Neurod4*, a proneural bHLH gene with a role in early neural lineage development, which functions downstream of *Neurog2* in embryonic cortical progenitors (reviewed in Oproescu et al., 2021). Notably, misexpression of *Neurod4* in P6-P7 astrocytes could similarly induce neuronal marker expression. However, in order for *Neurod4*-iNs to functionally mature, co-expression with *Insm1*, another commonly regulated TF, was required (Masserdotti et al., 2015). In support of these findings, a follow-up study using Ascl1 chromatin immunoprecipitation sequencing (ChIP-seq) and functional knockdown studies also identified *Neurod4* as an essential Ascl1 transcriptional target during the conversion of murine astrocytes to iNs (Rao et al., 2021). Additional essential downstream genes identified during this study included *Klf10*, *Chd7*, and *Myt1l* (Rao et al., 2021). Notably, another important finding of the Masserdotti et al. (2015) study was that astrocytes lose their capacity to undergo neuronal lineage conversion over time, which is due in part to increased activity of the repressor element 1 (RE1) silencing transcription factor (REST) repressive protein complex (Masserdotti et al., 2015). Notably, REST prevents *Neurog2* from activating *Neurod4* expression during iN lineage conversion (Masserdotti et al., 2015).

#### Metabolic Reprogramming Accompanies Astrocyte to iN Lineage Conversion

A major issue with direct reprogramming of P5-P7 astrocytes to iNs by the proneural TFs is that most astrocytes die within a few days post-transduction, except those few cells that make it to the iN fate more quickly (Gascon et al., 2016). To overcome this obstacle, Gascon et al. (2016) misexpressed *Ascl1* or *Neurog2* along with *Bcl2*, an anti-apoptotic gene that facilitated a more rapid rate of neuronal conversion, which caused more iNs to survive and mature (Gascon et al., 2016). Yet unexpectedly, the effect of *Bcl2* on neuronal lineage conversion was attributed to its role in reducing oxidative stress and a reduction in reactive oxygen species (ROS), and not its role in preventing apoptosis (Gascon et al., 2016). These studies raised the idea that aerobic metabolism, which produces ROS as a byproduct, may be an important consideration for reprogramming strategies.

Metabolic profiles of brain cells differ from one another—in general, NPCs in the developing brain favor glycolysis, while differentiated cells use oxidative phosphorylation (OXPHOS), which occurs in the mitochondria (Herrero-Mendez et al., 2009; Zheng et al., 2016). Mitochondria respond to stress by undergoing fusion with other mitochondria, while fission creates new mitochondria to adapt to an increase in energy demand. Interestingly, the proportion of mitochondria in each of these states differs between cortical astrocytes and neurons (Motori et al., 2013; Misgeld and Schwarz, 2017). In cortical astrocytes, mitochondria have elongated shapes consistent with mitochondrial fusion (Motori et al., 2013), while neurons exhibit smaller mitochondria, consistent with mitochondrial fission (Misgeld and Schwarz, 2017). Strikingly, *Ascl1*-iNs generated from murine P5 astrocytes also undergo a transition to a fission-like smaller mitochondrial morphology (Russo et al., 2020). Furthermore, LC-MS/MS of mitochondria from cortical neurons and astrocytes revealed a large number of differences, and a neuronal mitochondrial phenotype was recapitulated in *Ascl1*-iNs to varying degrees, depending on the extent of neuronal conversion (Russo et al., 2020). Finally, strikingly, the importance of differences in mitochondrial content was demonstrated by co-expressing *Ascl1* with neuronal-enriched antioxidant genes *Sod1* and *Prdx2*, which augmented astrocyte to iN lineage conversion (Russo et al., 2020).

Taken together, these studies demonstrate the capacity for lineage conversion between two neural lineages and highlight the striking capacity of the proneural bHLH TFs to specify neuronal subtype identities through the initiation of distinct neuronal programs.

### Direct Neuronal Reprogramming of Fibroblasts to iNs

#### *Brn2-Ascl1-Myt1l* (BAM)-Mediated Direct Neuronal Reprogramming

The term “direct neuronal reprogramming” was first coined in 2010 based on the lineage conversion of murine embryonic or postnatal fibroblasts into functional iNs through misexpression of a triple TF cocktail called the BAM or Wernig factors in honor of their discoverer (Figure 1). The BAM factors are *Brn2* (official gene name, *Pou3f2*), *Ascl1* and *Myt1l* (Vierbuchen et al., 2010). Unlike astrocytes, which can be converted to a neuronal identity by a single TF, optimal transdifferentiation of fibroblasts to iNs required three TFs in this study. This finding suggested that the barriers to iN reprogramming were less easily surmountable in fibroblasts, likely because the embryonic lineage is not neural, in contrast to astrocytes (Vierbuchen et al., 2010). While both excitatory and inhibitory postsynaptic currents could be evoked in BAM-iNs, most currents were excitatory (Vierbuchen et al., 2010). BAM gene expression could also be triggered in mouse embryonic fibroblasts (MEFs) using deactivated Cas9 fused to a transactivation domain (dCas9-VP64). dCas9-VP64 was targeted to BAM genes to initiate transactivation using guide RNAs (gRNAs) to each BAM gene (Black et al., 2016). Notably, this approach also generated iNs that could fire action potentials (Black et al., 2016). The ability to multiplex gRNAs makes this type of strategy attractive moving forward, especially to drive neuronal subtype identities that may require even more genes, as described further below.

In the interest of translating neuronal reprogramming technology to clinical practice, investigators turned their attention to human and non-human primate fibroblasts as a source cell type for lineage conversion. BAM TFs were shown to convert human fibroblasts isolated from 5.5- to 7-week-old fetuses to functionally active iNs (Pfisterer et al., 2011a, b). However, the efficiency of iN conversion was enhanced by co-expressing BAM TFs with the bHLH TF, *NEUROD1*, both in postnatal day (P) 1–3- or 8–10-week-old human fetal fibroblasts (Pang et al., 2011), and in embryonic skin fibroblasts from a non-human primate (marmoset) (Zhou et al., 2014). Interestingly, BAM-induced iN conversion could also be triggered *in vivo* after transplanting fibroblasts carrying doxycycline-inducible constructs into the striatum or hippocampus of adult rats, leading to iN integration *in vivo* (Torper et al., 2013).

There are striking differences reported in the efficacy of BAM TFs for neuronal reprogramming efficiency (Table 1). Reasons may be as simple as differences in culturing conditions and media components that alter the host fibroblasts. Moreover, fibroblast sources differ between studies, including human foreskin fibroblasts (Pang et al., 2011), or fibroblasts derived from fetuses after removal of the head, vertebral column, dorsal root ganglia, and internal organs (Pfisterer et al., 2011a, b). Indeed, a recent study has found that only a subset of mouse embryonic fibroblasts (MEFs) are amenable to reprogramming to an iPSC state, with cell competition resulting in the rapid domination by “elite clones” (Shakiba et al., 2019). Transcriptomic and epigenetic differences in MEFs that respond to overexpression of the Yamanaka factors have also been cataloged in great detail, with cells that do not become bona-fide iPSCs termed “F-class cells” (reviewed in Vidal et al., 2015). Further studies will be required to determine whether there are subsets of fibroblasts that are more easily converted to iNs by the usage of different TF combinations.

After the initial demonstration that BAM TFs could convert fibroblasts to iNs, there were efforts to reduce the number of required factors. Interestingly, overexpression of *ASCL1* alone can convert fibroblasts to excitatory iNs that make synapses, albeit not as efficiently, highlighting the importance of using all three BAM TFs (Vierbuchen et al., 2010; Chanda et al., 2014; Treutlein et al., 2016). Subsequent studies investigated the contributions of each of the BAM TFs to neuronal conversion. *Ascl1* was found to be a pioneer factor, as it opens the chromatin surrounding neural lineage genes, enabling Brn2 binding to chromatin transactivate downstream genes in neurogenesis (Wapinski et al., 2013). The ability of Ascl1 to open the chromatin occurs rapidly, within the first few days of the reprogramming process (Wapinski et al., 2017). The following 3 weeks of neuronal differentiation involves the functions of critical downstream TFs such as *Zfp238*, *Sox8*, and *Dlx3* (Wapinski et al., 2017).

In contrast to *Ascl1* and *Brn2*, *Myt1l* does not play a direct role in neurogenesis, but rather serves as a multi-lineage repressor that prevents the selection of alternative, non-neural lineages (Wapinski et al., 2013; Mall et al., 2017). In this regard, it is interesting that *Myod1*, a bHLH gene that normally specifies a skeletal muscle identity, but which binds to the same E-boxes as Ascl1, can induce the conversion of fibroblasts to iNs when co-expressed with *Myt1l* (Lee Q. Y. et al., 2020). *Myt1l* suppresses the activation of skeletal muscle genes so that the non-neural bHLH factor *Myod1* can convert fibroblasts to iNs (Lee Q. Y. et al., 2020).

The three BAM TFs thus each play critical roles in the neuronal reprogramming process. However, investigators have continued their quest to optimize reprogramming approaches, leading to additional critical insights into the underlying mechanisms of neuronal lineage conversion.

#### Other TF Combinations Confer Neuronal Subtype Identity Onto iNs

##### Dopaminergic iNs

After the seminal findings demonstrating BAM-mediated neuronal lineage conversion, several follow-up studies tested variations of the same theme. These studies used TF cocktails to generate iNs with the new goal of conferring different neuronal subtype identities. By generating iNs with specific neurotransmitter phenotypes, studies aimed to devise new strategies for neuronal repair of neurological disorders. For example, in Parkinson’s disease (PD), dopaminergic (DA) neurons in the nigrostriatal pathway are lost. Therefore, one of the first cell types targeted was DA neurons, for its therapeutic potential in the treatment of PD. A series of articles describing the generation of iDA neurons using direct neuronal reprogramming were first published in 2011. These studies described the conversion of fibroblasts to iDA neurons using a modification of the original Wernig strategy. The three BAM TFs were combined with *Lmx1a* and *Foxa2*, which specify a DA neuronal identity during development, to form a five TF cocktail that could convert human fibroblasts into induced DA neurons (iDAs) (Pfisterer et al., 2011a, b). Similarly, Ascl1 combined with *Nurr1* (official gene name, *Nr4a2*) and *Lmx1a*, a triple TF cocktail termed ANL, could convert mouse embryonic or adult tail tip fibroblasts, or human adult dermal or lung fibroblasts, into iDAs that had features of mature DA neurons (Addis et al., 2011; Caiazzo et al., 2011). A follow-up study of ANL-iDAs revealed that they could integrate in the striatum in a PD rat model, and improve motor behavior after DREADD-dependent activation (Dell’Anno et al., 2014).

Next, in an independent screen of eight TFs, a new combination of five TFs was identified; *Ascl1*, *Ngn2* (official gene name, *Neurog2*), *Sox2*, *Nurr1* (official gene name, *Nr4a2*), and *Pitx3*, which could reprogram human fibroblasts to iDA neurons (Liu et al., 2011). The transplantation of these cells into the striatum of a 6-hydroxydopamine (6-OHDA lesion) PD rat model also showed improved motor behavior (Liu et al., 2011). Using a similar screening strategy of 11 TFs, *Ascl1* and *Pitx3* were revealed to be capable of converting murine tail tip fibroblasts to an immature iDA fate, with the added expression of *Lmx1a*, *Nurr1* (official gene name, *Nr4a2*), *Foxa2* and *En1* leading to the expression of mature DA neuronal markers—a so-called six TF reprogramming cocktail (Kim et al., 2011b). This six TF cocktail also induced the generation of functional iDA neurons that could rescue motor behavior in 6-OHDA treated PD-like mice (Kim et al., 2011b). More recently, a three TF cocktail of *Brn2*, *Sox2*, and *Foxa1* was shown to convert murine fibroblast cells into DA precursor cells that could efficiently integrate and functionally differentiate into DA neurons in a MPTP lesioned PD mouse model after transplantation (Tian et al., 2015).

##### GABAergic iNs

An imbalance in excitatory-inhibitory circuits is a feature of many neuropsychiatric and epileptogenic disorders. Therefore, another neuronal type targeted for their neuronal repair potential has been GABAergic inhibitory neurons. In a screen of 21 TFs with known roles in specifying a GABAergic neuronal identity during development, a combination of five TFs was identified that could trans-differentiate MEFs into induced GABAergic neurons (iGABA-iNs): *Foxg1*, *Sox2*, *Ascl1*, *Dlx5*, and *Lhx6* (Colasante et al., 2015). *Ascl1*, *Dlx5*, and *Lhx6* have well-documented roles in the development of GABAergic neurons during embryogenesis. Conversely, the roles of *Foxg1* and *Sox2* were unexpected, but in this study, both TFs were shown to be required to facilitate *Ascl1* binding to the *Dlx1/2* enhancer (Colasante et al., 2015). The five TF-iGABA-iNs were transplanted into the adult murine hippocampus and shown to integrate functionally by optogenetic activation (Colasante et al., 2015).

##### Noradrenergic iNs

Several neural disorders related to sleep, arousal, and attention -hyperactivity disorder are linked to the dysfunction in the noradrenergic (NA) neurons (Benarroch, 2009). A seven-TF cocktail of TFs, namely *Ascl1*, *Phox2b*, *AP2a* (official gene name *Tfap2a*), *Gata3*, *Hand2*, *Nurr1* (official gene name *Nr4a2*), and *Phox2a*, could reprogram both murine fibroblasts and astrocytes into functional induced NA neurons (iNA) that can produce noradrenaline (Li et al., 2019). Strikingly, iNAs were electrically active, and when co-cultured with murine cardiac myocytes, they could induce myocyte beating, which provides strong support for their functionality (Li et al., 2019).

##### Motor Neurons

Similar to most parts of the adult mammalian brain, neurogenesis does not occur in the adult mammalian spinal cord to any significant extent. Finding new strategies to replace lost motor neurons has thus become a goal for the future of repair strategies and clinical therapeutic applications. The first demonstration that induced motor neurons (iMNs) could be generated *in vitro* involved the misexpression of the three BAM TFs with *Lhx3* (4 TF cocktail) and either *Neurog2*, *Hb9* (official gene name *Mnx1*) or all of these genes together with *Isl1* (5 TF cocktails) in adult mouse tail tip fibroblasts (Son et al., 2011). The 4TF- and 5TF- iMNs fired action potentials and formed neuromuscular junctions with chick myotubes (Son et al., 2011). Mazzoni et al. (2013) further refined this process to a triple TF cocktail: *Neurog2*, *Isl1*, and *Lhx3.* This triple TF cocktail was applied to mouse embryonic stem cells and induced differentiation into iMNs (Mazzoni et al., 2013). In this context, the authors were able to more carefully dissect apart the transcriptional events that underlie the acquisition of a MN identity, and revealed that there are two phases: firstly, *Neurog2*, *Isl1*, and *Lhx3* initially bind the same transcriptomic regions, followed by later differentiation events that are triggered by *Lhx3* and *Isl1* relocating to new regulatory sites in the genome (Velasco et al., 2017). To translate these findings to human fibroblasts, four TFs were shown to generate functional, cholinergic iMNs: *NEUROG2*, *SOX11*, *ISL1*, and *LHX3* (Liu et al., 2016). Interestingly, while *NEUROG2* alone combined with small molecules could induce the acquisition of a cholinergic iMN fate, mature MN markers (ISL1, LHX3) were not expressed (Liu et al., 2013). In contrast, the four TF-iMNs were electrically active and could form functional neuromuscular junctions (Liu et al., 2016).

Taken together, these studies demonstrate that a large number of different TFs can induce fibroblast to iN lineage conversion. This conclusion was further supported by a large-scale screen of 598 TFs, which identified 76 TF pairs that could generate iNs (Tsunemoto et al., 2018). Many starting paths can thus converge on the same final destination: the generation of iNs, as long as the developmental programs associated with neuronal differentiation are re-initiated. It seems fair to say from these findings, that once initiated, the neuronal differentiation program becomes self-propagating, at least to some extent. In this regard it is interesting that a CRISPR activation screen for inducers of neuronal differentiation in mouse embryonic stem cells (ESCs) identified a neuronal cell identity regulatory network that is maintained by several core factors (Liu et al., 2018). While technically not a directed neuronal differentiation study, this study supports the idea that there is not just one GRN that supports neurogenesis during reprogramming (Liu et al., 2018). However, given the evidence that the directed differentiation of ESCs into iGABA-iNs is further facilitated by the sequential expression of different TF modules (Au et al., 2013), the order in which these different network nodes are turned on is important, likely also in the reprogramming process.

#### miRNA Mediated Reprogramming of Fibroblasts to iNs

Additional advances in the direct neuronal reprogramming field were made with the inclusion of microRNAs (miR) into conversion cocktails (Figure 2). miRNAs are short non-coding RNAs that act as negative regulators of gene expression, either by promoting mRNA degradation or by blocking translation. Several miRNAs have been identified that promote neural lineage development by repressing the expression of genes that are not normally expressed in neural lineages (reviewed in Lang and Shi, 2012). miRNA technology can be incorporated to aid in the inhibition of alternative cell fates during cellular reprogramming. The first miRNA shown to aid direct neuronal reprogramming was miR-124, which was selected based on its abundant expression in the CNS and its role in attenuating the expression of several known negative regulators of neurogenesis, such as the poly-pyrimidine-tract-binding protein PTBP1, the neural progenitor (np) Brg/Brm-associated factors (BAF) complex, and REST (Ambasudhan et al., 2011; Xue et al., 2013). PTBP1 binds and regulates RNA biology in various ways. For instance, PTBP1 alters mRNA splicing and prevents miRNA activation through competitive binding or altering mRNA secondary structure (reviewed in Keppetipola et al., 2012). npBAF is a neural-specific SWI/SNF complex that is involved in chromatin remodeling, and overexpressing npBAF in fibroblasts can promote neuronal lineage conversion (reviewed in Staahl and Crabtree, 2013). Finally, REST forms a transcriptional repressor complex that is essential to silence neural lineage gene expression in non-neural cells (reviewed in Maksour et al., 2020). REST and miR-124 form a regulatory loop, with miR-124 repressing several genes in the REST complex, and REST repressing miR-124 expression (Xue et al., 2013).

Prior studies have revealed that overexpression of miR-124 may induce adult murine NPCs to differentiate into neurons (Cheng et al., 2009). To test whether miR-124 can aid direct neuronal reprogramming, a screen was performed by overexpressing miR-124 with different combinations of 11 TFs in human fibroblasts (Ambasudhan et al., 2011). This study identified *MYT1L*, *BRN2* (official gene name *POU3F2*), and miR-124 as the most efficient triple factor combination to induce the formation of electrically active iNs (Ambasudhan et al., 2011). As *ASCL1* was the only BAM component not in this reprogramming cocktail, it appears that at least some of *ASCL1’s* functions may be compensated for by miR-124.

In a similar study, miR-124 was combined with miR-9/9^∗^; the latter functions to represses *BAF53a* gene expression, a subunit of the npBAF complex involved in chromatin remodeling (Yoo et al., 2011). Subsequent studies identified downstream events that underlie miR-124 and mir-9/9^∗^ neuronal lineage conversion, and identified two sequential steps: (1) silencing of the TFs *KLF4* and *KLF5*, to repress a fibroblast fate, and (2) upregulation of *RN7SK*, a small nuclear RNA required to activate neuronal lineage genes through changing chromatin accessibility (Cates et al., 2021). The importance of the epigenome in reprogramming is discussed further below.

TFs and other miRs were also combined with miR-124 and mir-9/9^∗^ to further improve the conversion of human fibroblasts to iNs. For example, the combined misexpression of miR-302/367, a cluster containing five miRNAs that also facilitates reprogramming to generate iPSCs, with miR-124 and miR-9/9^∗^, enhances neuronal lineage conversion (Zhou et al., 2015). Similarly, co-expression of miR-124 and mir-9/9^∗^ with *ASCL1* could convert fibroblasts from other species, such as adult porcine and marmoset fibroblasts, into iNs (Habekost et al., 2020; Nemoto et al., 2020). However, the conversion of human fibroblasts from neonatal foreskin or adult dermis to iNs was most efficient when miR-124 and miR-9/9^∗^ were combined either with *NEUROD2*, or with both *ASCL1* and *MYT1L* (Yoo et al., 2011).

Overexpression of miRs has also been incorporated into neuronal reprogramming strategies that are targeted toward neuronal subtype specification. For example, co-expression of miR-124 and miR-9/9^∗^ with *CTIP2* (official gene name, *BCL11B*), *DLX1*, *DLX2*, and *MYT1L* (termed *CDM*) effectively transformed human fibroblasts into induced medium spiny neurons (iMSNs) (Victor et al., 2014). Of promise, transplantation of the iMSNs into the striatum, where these neurons are normally found, led to successful engraftment and integration into the host neural network (Richner et al., 2015). Similarly, co-expression of miR-124 and miR-9/9^∗^ with *ISL1* and *LHX3* promoted the differentiation of iMSNs that possessed subtype-specific transcriptomic and epigenomic profiles (Abernathy et al., 2017).

Given the capacity of miRNAs to promote neuronal lineage conversion, it is not surprising that an upstream regulator, *PTB* (official gene name, *PTBP1*), can also influence neuronal reprogramming (Xue et al., 2013). The knockdown of *PTBP1* converted MEFs to iNs by inhibiting the ability of REST to repress neural lineage gene expression, and by regulating mRNA splicing and stability (Xue et al., 2013). Conversely, *PTBP1* knockdown in human adult fibroblasts (HAFs) could not promote lineage conversion to a mature neuronal phenotype, unless it supported by the additional knockdown of another PTB paralog, *nPTB* (official gene name, *PTBP2*). In human cells, *PTBP2* knockdown leads to expression of *BRN2* and miRNA-9 which are both critical determinants of neuronal differentiation and neuronal reprogramming, as highlighted above (Xue et al., 2016).

As a final note, the ability of miR-124 to repress neuronal gene expression has also been exploited in direct neuronal reprogramming to deactivate the reprogramming factors after they acquire an iN identity (Lau et al., 2014). By linking four copies of the miR-124 target sequence to *ASCL1*, *BRN2*, and *MYT11*, the authors were able to demonstrate that upon the conversion of human fibroblasts to iNs, the expression of these conversion factors were silenced (Lau et al., 2014). This type of approach is more amenable to human therapies, because if high levels of transgene expression is not maintained, the risk of tumor formation is reduced.

#### Epigenetic Changes Drive Fibroblast to iN Reprogramming

Cellular reprogramming involves the shutdown of GRNs associated with the starting somatic cell type, and the activation of new gene expression profiles associated with the reprogrammed cell type. These changes are tightly linked to chromatin opening and closing so that the appropriate target genes are accessible to lineage specifying TFs. However, the process of lineage conversion faces the unsurprising challenge that some starting somatic cell populations retain their cell-type specific gene regulatory networks. For instance, iNs reprogrammed from fibroblasts continued to retain some fibroblast genes, a finding that was evident in a large-scale screen of 598 TFs that identified 76 TF pairs that could induce neuronal reprogramming (Tsunemoto et al., 2018). Understanding how chromatin dynamics contribute to incomplete lineage conversion is therefore important.

To begin with the investigation of iNs reprogrammed from fibroblasts, several bHLH TFs have been identified with pioneering activity (e.g., *Ascl1*), which means that they can bind to and facilitate the opening of closed chromatin so that other neurogenic TFs (e.g., *Brn2*) can bind target sites (Wapinski et al., 2013). Similarly, *Neurod1* binds and rearranges closed chromatin in target regions during microglia to iN conversion, especially those sites with bivalent trimethylation, which is a feature of genes that are poised for transcription, as described below (Matsuda et al., 2019). In contrast, Neurog2 cannot act as a pioneer factor unless combined with two small molecules, forskolin, and dorsomorphin, which help to remove barriers to chromatin binding (Smith et al., 2016).

Unlike these pioneer TFs, most other TFs require that the chromatin surrounding their target sites are first opened before they can bind. A new concept emerging in cellular reprogramming is that epigenetic regulators act as gatekeepers that maintain terminally differentiated cell fates which in turn prevent lineage conversion (Gascon et al., 2017b). Gatekeepers control chromatin “opening” (accessible) and “closing” (inaccessible) to regulate the binding of lineage-specifying TFs that initiate differentiation programs. The status of chromatin (i.e., open or closed) is controlled by three main events: (1) histone modifications, (2) DNA methylation and (3) nucleosome remodeling, the modulation of each of which, can influence neuronal reprogramming, as discussed below.

##### Histone Modifications

The most extensively studied histone modifications involve the acetylation or methylation of specific lysine residues (K) in histone subunits H3 or H4. Changes are commonly found in the regulatory regions of actively transcribed genes, including H3K9 acetylation (ac), or the addition of one (me1), two (me2) or optimally three (me3) methyl groups to H3K4 (H3K4me1-3) at transcription start sites. Conversely, H3K36 (H3K36me1-3) methylation is found in the body of actively transcribed genes. The chromatin marks associated with gene silencing include H3K9me3, H3K27me3, and monoubiquitinylation of H2AK119ub. Lineage gatekeepers primarily act *via* the polycomb repressive complexes 1/2 (PRC1/2), which compact chromatin by catalyzing the repressive H3K27me3 and H2AK119ub modifications (Tursun et al., 2011; Cheloufi et al., 2015; Gascon et al., 2017a).

Chromatin changes have been most extensively studied during reprogramming to pluripotency to generate iPSCs. H3K9me3 acts as a barrier to iPSC reprogramming, such that the overexpression of histone demethylases (e.g., Jmjd2c) or the knockdown of histone methylases (e.g., G9a, Setdb1) specific to H3K9me3 aids reprogramming (reviewed in Stricker and Gotz, 2021). Similarly, H3K27me3 marks on neuronal lineage genes are among the earliest changes to occur when microglia are reprogrammed to iNs by *Neurod1* (Matsuda et al., 2019). A loss of H3K4 methylation occurs in genes that maintain the starting somatic cell state early on during the iPSC reprogramming process (reviewed in Stricker and Gotz, 2021).

In some instances, both activating (H3K4me3) and repressive (H3K27me3) chromatin modifications can co-occur in the regulatory regions of genes that are “poised” for transcriptional activation (Bernstein et al., 2006). To facilitate the activation of adult NSCs, Sox2 binds to “poised” genes, limiting PRC2 activity at these bivalent sites to reduce H3K27me3 modifications and increase H3K9ac marks at critical neurogenic loci, such as at *Neurog2* and *Neurod1* (Amador-Arjona et al., 2015). It is intriguing to speculate that a similar role for SOX2 may be found during the reprogramming of fibroblasts to iNs. Indeed, SOX2 augments *Neurog2*- or *Ascl1*-mediated reprogramming of human umbilical cord mesenchymal stem cells into iNs (Araujo et al., 2018), and is included in conjunction with proneural genes and other TFs to form iGABA neurons (Colasante et al., 2015) and iDA neurons (Liu et al., 2011).

Ascl1 preferentially binds a different trivalent mark during BAM-induced MEF to iN reprogramming; H3K27a, H3K4me1, and H3K9me3 (Wapinski et al., 2013). It is surprising that the H3K9me3 mark, which normally represses iPSC reprogramming, is required for Ascl1 binding and iN lineage conversion, since overexpression of Jmjd2c, which removes this mark, reduces neuronal conversion efficiency (Wapinski et al., 2013).

While there remains much to be sorted out in terms of understanding the contribution of histone modification to neuronal lineage conversion, it is clear that these changes play a fundamental role in cellular reprogramming.

##### DNA Methylation

DNA is mainly methylated on cytosines by DNA methyltransferases (DNMTs), primarily in CpG dinucleotides that are located in regulatory regions of the genome. To a smaller extent DNA can also be methylated on non-CpG sequences (termed CpH), which are often found in non-regulatory regions of the genome. Strikingly, CpH methylation is most abundant in mature mammalian neurons and not frequently found in other cell types (Guo J. U. et al., 2014). Methylated cytosines repress transcription by closing chromatin through the recruitment of methylated-DNA binding domain (MBD) proteins, that recruit additional factors to confer a repressive chromatin structure. The removal of the repressive DNA methyl groups is achieved by ten-eleven translocation (TET) methylcytosine dioxygenases. Recent studies have revealed that the DNA methylome is dynamically regulated during BAM-induced MEF to iN reprogramming (Luo et al., 2019). Strikingly, *Ascl1* also displays pioneer functions in this regard, through inducing CpH methylation in a neuron-like signature in iNs, which is a DNMT3A dependent process (Luo et al., 2019).

##### Chromatin Remodeling

Nucleosome remodeling involves ATP-dependent nucleosome movements that are facilitated by altering how DNA interacts with histones, which can result in opening of the chromatin to make it accessible for TF binding. The main regulators of chromatin remodeling are different BAF (Brg1/Brm-associated factor) complexes, also known as a SWI/SNF complexes, which disassemble nucleosomes to convert heterochromatin to euchromatin. Surprisingly, a role for chromatin remodeling in neuronal reprogramming was revealed in β-actin (official gene name, *Actb*) KO mice (Xie et al., 2018). *Actb* KO MEFs have a reduced capacity to undergo neuronal lineage conversion because Brg1, a BAF subunit, cannot bind chromatin, which results in high levels of sustained H3K9 and H3K27 methylation and consequent chromatin compaction (Xie et al., 2018).

### Small Molecules Aid *in vitro* Neuronal Reprogramming

As expected, media composition is critically important for all *in vitro* neuronal reprogramming strategies and has roles that include supporting iN survival and augmenting the effects of overexpressing neural TFs and miRNAs. As such, in parallel to tests of intrinsic factors, investigators have been working on generating defined media to promote neuronal lineage conversion without the requirement to express exogenous genes or miRNAs. Critical studies that highlight the impact of media components on neuronal reprogramming are summarized in Table 2 (for murine studies) and Table 3 (for human studies). Here we simply highlight the rationale for choosing various factors.

**TABLE 2:** Small molecules used in iN lineage conversion strategies in rodent cells.

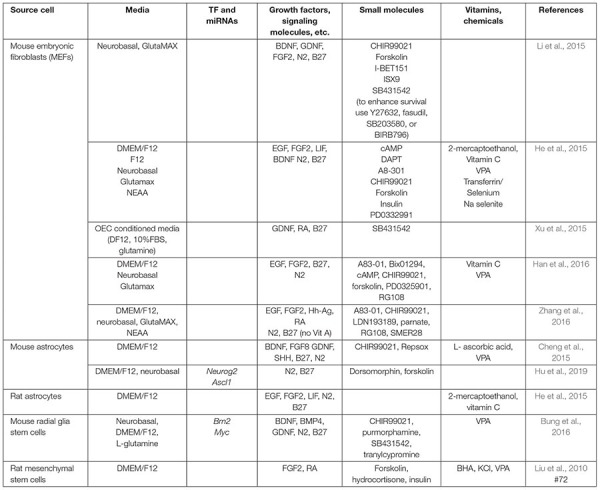

**TABLE 3:** Small molecules used in iN lineage conversion strategies in human cells.

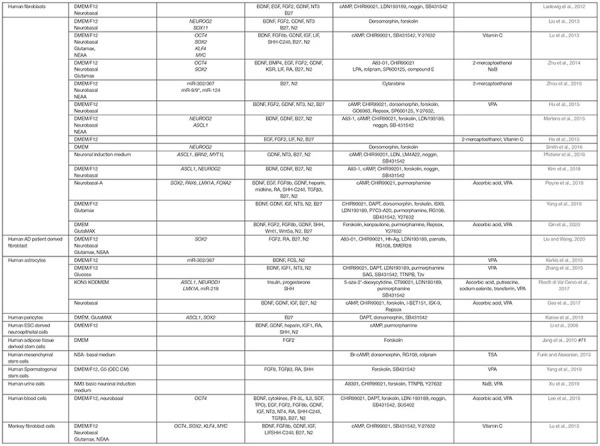

**FIGURE 3:**
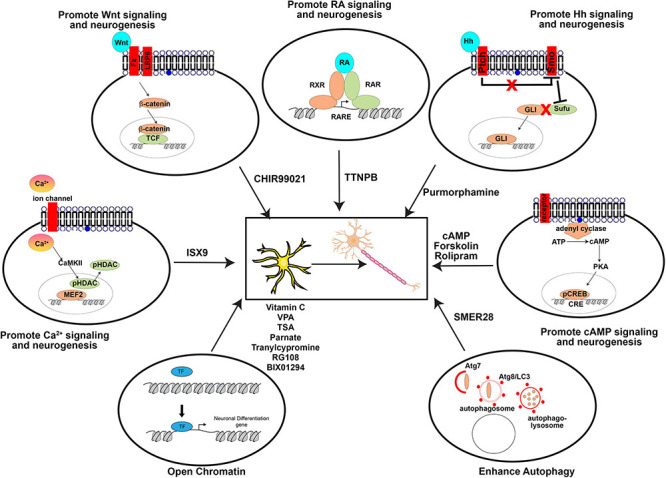
Pathways activated by small molecules to aid direct neuronal reprogramming. A summary of the agonists/antagonists used in neuronal reprogramming cocktails that activate critical signaling pathways or cellular processes.

**FIGURE 4:**
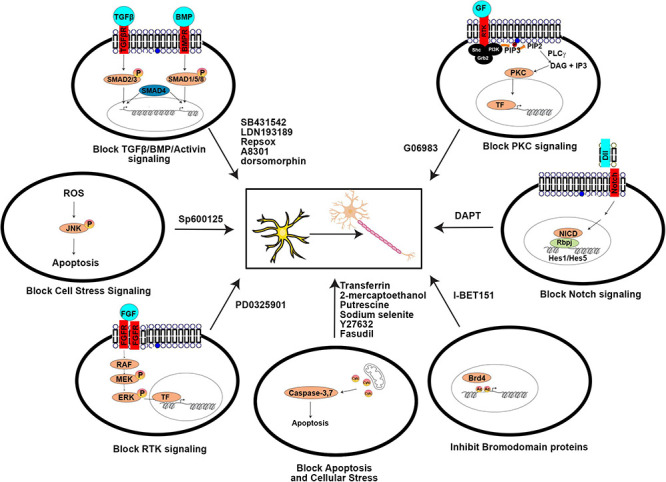
Signaling pathways blocked by small molecules to aid direct neuronal reprogramming. A summary of the agonists/antagonists used in neuronal reprogramming cocktails that inhibit critical signaling pathways or cellular processes.

As with the direct manipulation of gene expression, media design makes the same considerations of which pathways to activate to promote neuronal differentiation, and to inactivate or prevent the acquisition of alternative cell fates. Factors added to culture media can be roughly considered in three groups: (1) growth factors, supplements, cytokines, and other signaling molecules, (2) small molecule agonists or antagonists of signaling molecules, and (3) other vitamins or chemicals that impact cellular status (pH, reducing capacity, etc.). In general, these molecules/chemicals either turn on (summarized in Figure 3) or turn off (summarized in Figure 4) pathways to promote neuronal lineage conversion.

#### Growth Factors, Supplements, Cytokines, and Other Signaling Molecules

The most common growth factor added to cell culture media are fibroblast growth factors (FGF2/4/8), epidermal growth factor (EGF) or insulin growth factor (IGF), which support general cell proliferation, along with N2 and/or B27 defined growth supplements that supports neuronal growth and survival. Other growth factors are added because they promote neuronal differentiation, such as Wnts, retinoic acid, sonic hedgehog (SHH), and the sex steroid, progesterone (reviewed in Rivetti di Val Cervo et al., 2017; Oproescu et al., 2021).

#### Small Molecule Antagonists

Several signaling pathways are targeted for shutdown by small molecule antagonists for neuronal reprogramming, as described below:

##### Activin/BMP/TGFβ/ALK Signaling

Signaling through these TGFβ family pathways specifies mesoderm and endoderm lineages and must be inhibited to allow neural fate specification (Chambers et al., 2009). Notably, BMP signaling also promotes Ascl1 degradation and therefore promotes gliogenesis and inhibits neurogenesis (Vinals et al., 2004). Inhibitors of these pathways include SB431542, LDN193189, dorsomorphin, RepSox, and A8301.

##### Notch Signaling

Notch binds Jag/Dll ligands on adjacent cells, ultimately leading to the downstream transcription of *Hes1/Hes5*, bHLH TFs that inhibit proneural gene expression and prevent neuronal differentiation. Repression of Notch signaling is achieved using DAPT (N-[N-(3,5-difluorophenacetyl)-l-alanyl]-S-phenylglycine t-butyl ester), a gamma secretase inhibitor that prevents cleavage of the Notch receptor so that the Notch intracellular domain (NICD) does not translocate to the nucleus to assist in the transcriptional activation of the downstream *Hes1/Hes5* bHLH effectors (reviewed in Oproescu et al., 2021).

##### Bromodomain Binding Proteins

Bromodomain proteins bind acetylated proteins, such as histones. I-BET151 is a BET bromodomain inhibitor that prevents these proteins from binding their targets, with diverse effects, including cytokine-induction of downstream STATs.

##### Cell Death Pathways

Antagonists such as ROCK inhibitors (e.g., Y27632, fasudil; Li et al., 2015) are also used to promote cell survival during neuronal lineage conversion. Furthermore, using a P7C3 or a P7C3-A20 derivative may be helpful as they are nicotinamide phosphoribosyl transferase (NAMPT) that have neuroprotective effects promoting survival (Yang et al., 2019).

##### Other Signaling Pathways

Inhibition of other signaling pathways has also been found to improve the conversion of fibroblasts to iNs, including: PKC signaling, which is blocked with G06983, JNK signaling, which is blocked with SP600125, p38MAPK signaling, which is blocked with SB203580 or BIRB796, and MEK signaling, which is blocked with PD0325901 (Han et al., 2012; Hu et al., 2015; Li et al., 2015).

##### Epigenetic Modifiers

Several factors that open chromatin have been used in neuronal reprogramming, with the rationale that they will facilitate binding of lineage-specifying TFs to their target sites. Included are inhibitors of DNA methylation, such as 5-azacytidine or 5-aza-2’-deoxycytidine. Similarly, inhibitors of DNA methyltransferase (DNMT), namely RG108 or BIX01294, can prevent DNA methylation, which also opens up the chromatin for TF binding. Tranylcypromine is an inhibitor of lysine-specific demethylase 1 (LSD1), which converts H3K4me2 to H3K4me1 or H3K4me0 by removing a chromatin mark associated with open chromatin used in neuronal reprogramming (Bung et al., 2016). Similarly, parnate is also a histone demethylase inhibitor used in neuronal reprogramming (Zhang et al., 2016).

#### Small Molecule Agonists

Rather than adding signaling molecules directly, small molecules can also be used to activate pathways that promote neuronal differentiation. Neuronal differentiation pathways that have been targeted with small molecules include:

##### Wnt Signaling

Wnts promote embryonic neurogenesis (Li et al., 2012), the activation of which is achieved using a small molecule that inhibits GSK3 activity (e.g., CHIR99021), as GSK3 is a downstream negative regulator of WNT signaling.

##### Cyclic-AMP (cAMP) Signaling

Activation of adenyl cyclase converts ATP to cAMP, which activates PKA, leading to phosphorylation of cAMP response element binding (CREB) protein (Montminy and Bilezikjian, 1987), which then translocates to the nucleus to initiate transcription and neuronal differentiation (Shan et al., 2008). This pathway can be achieved by adding cAMP directly, or using forskolin (Jang et al., 2010). In addition, rolipram can activate cAMP by inhibiting type 4 cyclic nucleotide phosphodiesterases (PDE4) (Sasaki et al., 2007).

##### Retinoic Acid (RA) Signaling

RA is a potent activator of neurogenesis, and acts as a ligand for nuclear receptors of the RXR and RAR TF family, which dimerize to bind RARE elements in the genome (Diez del Corral et al., 2003). RA signaling can be activated *in vitro* using TTNPB (Maloney et al., 2000).

##### Hedgehog (Hh) Signaling

Hh binds the Ptch receptor, which then releases its inhibitory control of Smo. Once Smo is activated, it represses Sufu, releasing Sufu inhibition of the Gli TFs. Thus, as a consequence of Hh activation of Ptch, Gli TFs can translocate to the nucleus and activate transcription. Hh signaling is activated by purmorphamine, which binds the Smo receptor to facilitate neuronal differentiation and reprogramming (Li et al., 2008).

##### Calcium-Dependent Signaling

Ca^2+^ enters the cell through ion channels, and once intracellular Ca^2+^ concentrations increase, CaMKII is activated. CaMKII can phosphorylate HDACs, leading to their translocation out of the nucleus, to allow different TFs to promote neurogenesis, such as MEF2. Once HDAC interactions are removed, these TFs then transactivate neurogenic genes. ISX9 induces neural lineage development by activating calcium dependent signaling (Petrik et al., 2012).

##### Autophagy

Autophagy sustains cellular homeostasis by removing damaged organelles so that they can be replaced by new healthy organelles. Autophagy begins with Atg7, which acts like an E1 activator protein for ubiquitin ligases, which helps to “mark” damaged organelles, and activates Atg8/LC3. Atg8 is then required followed by the sequential formation of autophagosomes and then autophagolysosomes. Autophagy can be induced in an mTOR-independent fashion using SMER28, which enhances neuronal reprogramming (Zhang et al., 2016).

#### Other Vitamins or Chemicals

##### Vitamin C (Ascorbic Acid)

Vitamin C is added to reprogramming cocktails not only for its role as an antioxidant, but also for its activity as a modulator of the epigenome (Lee Chong et al., 2019). Vitamin C increases the activity of the Jumonji-C domain histone demethylases (JHDMs), which remove repressive H3K9 and H3K27 marks from chromatin. Vitamin C also increases the activity of ten-eleven translocation (TET) family DNA hydroxylases which “erase” DNA methylation (Lee Chong et al., 2019). Thus, vitamin C facilitates somatic cell reprogramming, both for iPSCs and for iNs, by opening up the chromatin to make it accessible for TF lineage determinant (Esteban and Pei, 2012; Lee Chong et al., 2019).

##### HDAC Inhibitors

HDAC inhibitors include valproic acid (VPA), sodium butyrate (NaB) and trichostatin A (TSA), prevent the removal of acetyl groups from histones. This therefore confers a generally open chromatin structure due to electrostatic repulsion between negatively charged DNA and acetylated histone groups. For example, NaB facilitates somatic cell reprogramming to an iPSC fate through preventing *Oct4* reprogramming TF from associating with HDACs (Zhang et al., 2014). Consequently, *Oct4* can elevate expression of the miR-202/367 cluster (Zhang et al., 2014) and is also important for neuronal lineage conversion (Zhou et al., 2015).

##### Other Chemical Agonists

Putrescine is a diamine that controls cellular stress response, regulates transcription, promotes stem cell self-renewal (Zhao et al., 2012), and enhances neuronal reprogramming (Rivetti di Val Cervo et al., 2017). 2-mercaptoethanol, which is a reducing agent, is also added to media during reprogramming as a potent reducing agent that removes free radicals, thereby preventing cell death. Transferrin binds iron, which reduces iron-induced cell death of iNs (Cozzi et al., 2019). Sodium selenite inhibits ROS-mediated apoptosis of neural cells (Yeo and Kang, 2007).

### Neuronal Reprogramming Involving a Neural Precursor Cell or Neural Stem Cell Intermediate

Two major neural trans-differentiation approaches are being used: direct transformation of source cells into iNs, as described above, or the prior conversion of somatic cells to an induced neural progenitor cell (iNPC) or induced NSC (iNSC) state before the induction of neuronal differentiation. The advantage of first generating iNPCs or iNSCs is that these cells are proliferative and can be expanded before terminal differentiation to non-expandable iNs.

The first protocol developed to generate iNPCs from MEFs involved the transient expression of the four Yamanaka factors (*Oct4*, *Sox2*, *Klf4*, *Myc*) with the addition of FGF2/4 and EGF to support iNPC proliferation (Kim et al., 2011a). Culturing iNPCs in media containing an N2 supplement promoted neuronal differentiation, yielding functional iNs that invoke action potentials (Kim et al., 2011a). Importantly, with this paradigm, the MEFs did not pass through a pluripotent iPSC state, which reduces the risk of tumorigenicity (Kim et al., 2011a). Since then, efforts were also made to introduce the four Yamanaka TFs in an integration free manner into human and monkey fibroblasts using a Sendai virus that could be inactivated by heat (39°C) (Lu et al., 2014). In this study, small molecules such as LIF, SB431542 (to block TGFb) and CHIR99021 (to block GSK3 and activate Wnt) were also included (Lu et al., 2014). Human neonatal dermis-derived fibroblasts or adult adipose-derived stem cells have also been converted to an iNSC identity by misexpressing the four Yamanaka TFs and growing on a MEF feeder layer (Cairns et al., 2016). When the MEF feeder layer was later removed this allowed for the differentiation of human iNSCs into neuronal and glial cells that were successfully engrafted into either an embryonic chick brain and in a 3D human brain tissue model (Cairns et al., 2016). Finally, misexpression of the BAM factors together with *Bcl-xl* in murine embryonic fibroblasts or adult tail tip fibroblasts converts fibroblasts to an intermediate iNPC identity (Lim et al., 2015). Neuronal differentiation can then be achieved in a second step through the misexpression of TFs such as *Nurr1* and *Foxa1*, which confer a dopaminergic neuronal identity (Lim et al., 2015).

Several groups have also subtracted some of the Yamanaka TFs and were still able to generate iNPCs/iNSCs. For example, by expressing the four Yamanaka TFs but restricting *Oct4* expression to the initial reprogramming phase, mouse fibroblasts could form iNSCs that had the capacity to self-renew for >50 passages, a hallmark feature of stem cells (Thier et al., 2012). Similarly, the expression of *OCT4*, *NANOG*, or *SOX2* in human astrocytes generated iNPCs, that could be differentiated into iNs *in vivo* through the additional expression of *ASCL1* (Corti et al., 2012). The expression of only *Sox2*, *Klf4*, and *Myc* together with *Brn4* (official gene name, *Pou3f4*) and *Tcf3* could also induce mouse fibroblasts to a self-renewing iNSC identity (Han et al., 2012). In another study, self-renewing iNSCs were generated by the misexpression of a single Yamanaka factor, *Sox2*, in murine and human fibroblasts (Ring et al., 2012). Similarly, misexpression of *OCT4* alone could induce human fibroblasts to transdifferentiate into iNPCs with trilineage potential (Mitchell et al., 2014). In a similar study, *OCT4* overexpression was combined with several small molecules (CHIR99021, A83-01, sodium butyrate, LPA, rolipram, SP600125), termed cell activation and signaling-directed (CASD), to improve the conversion of human fibroblasts to iNSCs (Zhu et al., 2014). However, there is some concern for all the Yamanaka factors in the use of neuronal reprogramming due to their tumorigenic potential. For example, *Oct4* misexpression can lead to the formation of dysplastic lesions (Hochedlinger et al., 2005), suggesting that this type of approach may not be suitable for clinical applications.

To overcome the low reprogramming efficiency from fibroblast into iNSCs, *Sox2* can be used in addition to *Klf4*, *Myc* and *Brn4*, as these factors are known to reprogram fetal fibroblast into neural restricted precursor cells (Kim et al., 2014; Zou et al., 2014). In a subsequent screen of 11 neural TFs, the combination of *Sox2*, *Brn2*, and *Foxg1* was found to be sufficient to convert murine fibroblasts to iNPCs with trilineage potential (Lujan et al., 2012). A screen of 14 TFs identified a panel of eight TFs (*Brn2*, *Hes1*, *Hes3*, *Klf4*, *Myc*, *NICD*, *Plagl1*, *Rfx4*) that can convert not only MEFs, but also adult liver cells and B lymphocytes into iNSCs (Cassady et al., 2014). In another expanded screen of 25 TFs, both 6 TF and 7 TF combinations were identified that could convert human fibroblasts into iNPs (Hou et al., 2017). Thus, as with programs to generate iNs directly, there are a variety of different molecular cocktails that can be used to generate iNSCs or iNPCs.

### Direct Neuronal Reprogramming of Other Somatic Cell Types to iNs

While initial studies on direct neuronal reprogramming used either astrocytes or fibroblasts as starting cell types, a number of groups have begun to explore the possibility of converting other somatic cell types to iNs, including brain pericytes, olfactory ensheathing cells (OECs), adipocyte progenitor cells, hepatocytes, bone marrow derived mesenchymal stem cells, microglia, and others. Technically, some of these protocols should be classified as induced differentiation rather than lineage conversion strategies as they began with cells in a progenitor cell state, but as they involve the interconversion of one cell lineage to another, they are described herein.

The use of the BAM TFs, either alone or in combination with other factors, has been applied to the reprogramming of other somatic cell types in addition to fibroblasts with great success. Subsequent studies confirmed that BAM TFs could also trans-differentiate other murine somatic cell sources into iNs, including adipocyte progenitor cells (Yang et al., 2013) and hepatocytes, both from early postnatal mice, with the latter study further confirming silencing of the hepatocyte transcriptome (Marro et al., 2011). In another study, BAM TFs were combined with *Neurod1* and were shown to convert murine and human iPSC-derived cardiomyocytes, which are a mesodermal lineage, to iNs that could fire action potentials (Chuang et al., 2017). In a similar study, vascular pericytes isolated from the human brain were transdifferentiated into iNs using BAM TFs along with *Tlx3* and miR-124 (Liang X. G. et al., 2018). Interestingly, in pericytes, *Myt1l*, one of the BAM components, was sufficient on its own to promote pericyte to iN conversion, and it did so in part by elevating the expression levels of *Ascl1*, *Brn2*, and *Neurog2* (Liang X. G. et al., 2018). Given that *Myt1l*’s role in neuronal lineage conversion has been attributed to its role in turning off the expression of non-neural genes, this finding raises the intriguing possibility that neural lineages are somehow a default fate of brain pericytes. Taken together, these studies support the robustness of the BAM cocktail for neuronal lineage conversion of a vast array of cell types.

Neuronal reprogramming of other somatic cell lineages has also been achieved using some of the original Yamanaka factors. For instance, *Sox2*, a SoxB1 TF that is both used to generate iPSCs and is a critical determinant required for specifying and maintaining a NPC identity during development has been primarily used by investigators (Zhang and Cui, 2014). In one study, *SOX2* was misexpressed along with *MYC*, also one of the original Yamanaka factors, in CD133 positive human cord blood cells, resulting in the formation of iNs that were functionally active (Castano et al., 2014). Notably, the same conversion strategy was less efficient at converting peripheral blood mononuclear cells into functionally active iNs, highlighting intrinsic differences in the responses of different host cells to reprogramming factors (Castano et al., 2014). Similarly, *Oct4* was used to transform cord blood and adult peripheral blood cells to iNPCs but required the addition of small molecules inhibitors of SMAD and GSK3 signaling (Lee et al., 2015), contrasting to fibroblasts in which *Oct4* alone was sufficient for neuronal lineage conversion (Mitchell et al., 2014). Notably, blood-derived iNPCs differentiated into astrocytes or glutamatergic excitatory iNs (and not oligodendrocytes) after xenografting into the murine brain and could also be directed to a dopaminergic identity if pre-treated with Shh and FGF8b (Lee et al., 2015).

*SOX2* was also combined with the proneural gene *ASCL1* to transdifferentiate pericytes isolated from the human brain into functionally active iNs (Karow et al., 2012). However, in a follow-up study using scRNAseq to study lineage trajectories, the transdifferentiation of human pericytes to iNs was shown to traverse a NSC-like intermediate state, arguing that this procedure is not technically “direct” neuronal reprogramming (Karow et al., 2018). Similarly, *Sox2*/*Ascl1* and *Sox2*/*Neurog2* combinations were demonstrated to convert human umbilical cord mesenchymal stem cells (UMSC) to electrically active iNs (Araujo et al., 2018). Interestingly, when *Neurog2* was expressed in rat mesenchymal stem cells (MSCs) that were transplanted into the striatum 1 day post-middle cerebral artery occlusion (MCAO) stroke in rats, the MSCs transformed into a NPC-like intermediate, and then transdifferentiated into electrically active iNs that could facilitate behavioral recovery (Cheng et al., 2014). As described further below, as the MSCs went through a NPC intermediate, it is not technically direct reprogramming, suggesting that *Neurog2* alone is not sufficient to directly promote lineage conversion, at least in this non-neural starting cell type, without a progenitor intermediate (Cheng et al., 2014).

Another source of somatic cells that are “readily available” for reprogramming are olfactory ensheathing cells (OECs), a glial cell type in the olfactory epithelium (Sun et al., 2019). Interestingly, OECs can be converted to iNs by the expression of *Neurog2* alone, which yields iNs that generate action potentials, form synapses, and integrate into the adult spinal cord (Sun et al., 2019). Given that glial cells are also neural, it is perhaps not surprising that OECs can be converted to iNs with one proneural TF. Indeed, conditioned media from OECs can induce the differentiation of spermatogonial stem cells into dopaminergic neurons when combined with other small molecules in a defined media (Yang et al., 2014). Early postnatal murine Sertoli cells could also be transdifferentiated into iNSCs using a cocktail of nine TFs (Ascl1, *Neurog2*, *Hes1*, *Id1*, *Pax6*, *Brn2*, *Sox2*, *Myc*, *Klf4*), which display trilineage potential, generating astrocytes, oligodendrocytes and neurons that are dopaminergic, GABAergic, or cholinergic (Sheng et al., 2011).

Neuronal reprogramming has also been applied to the inner ear, with the goal of replacing primary auditory neurons, which is achieved by the expression of *Ascl1*, or *Ascl1* together with *Neurod1* in cochlear non-sensory epithelial cells to form iNs that have the phenotype of auditory neurons (Nishimura et al., 2014). Notably, *Neurod1 and Ascl1* were also co-expressed in spiral ganglion non-neuronal cells to generate iNs that had a transcriptomic profile that resembled primary auditory neurons, and which could project to cochlear hair cells in co-cultures *in vitro* (Noda et al., 2018).

Strikingly, in a screen of eight TFs, including all of the BAM TFs, *Sox2*, *Neurog2*, and *Neurod1*, as well as miR-124 and *MBD3*, an epigenetic modifier, misexpression of *Neurod1* alone was found to be sufficient to convert murine microglial to iNs (Matsuda et al., 2019). Notably, in this study, *Neurod1* acts as a pioneer factor, binding to and opening closed regions of the chromatin, to facilitate neuronal lineage conversion (Matsuda et al., 2019).

In summary, a variety of different somatic cell types can be converted to iNs, with different TF combinations active depending on the starting cell type. One of the important revelations from these studies was that in some instances, somatic cells transit through a NPC or NSC intermediate stage.

## Applications for Induced Neurons Generated *in vitro via* Direct Neuronal Reprogramming

### Important Considerations for Disease Modeling Using Patient-Derived iNs

**FIGURE 5:**
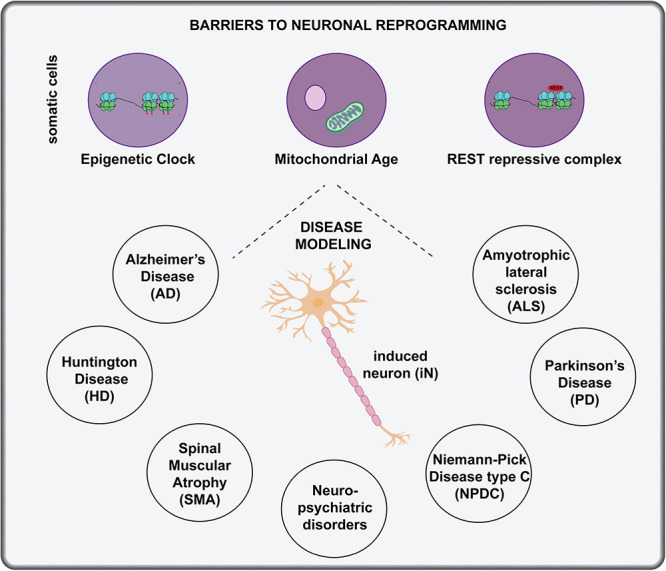
Applications of *in vitro* direct neuronal reprogramming. The generation of iNs in a dish has great potential for disease modeling, but several considerations must be made when using host cells from aged individuals, including changes to the epigenetic clock, mitochondrial age and activity of the REST repressive complex. Diseases that have been modeled with *in vitro* neuronal lineage conversion beginning with patient cells include AD, ALS, PD, HD, Niemann-Pick disease type C, and neuro-psychiatric disorders.

One of the main applications for patient-derived iNs is modeling of neurological disease and neurodevelopmental disorders, including those with genetic or environmental causes. Several considerations must be taken into account to optimize lineage conversion for disease modeling, especially when somatic cells are reprogrammed from elderly patients (Figure 5):

#### Epigenetic Clock

Direct reprogramming to generate iNs has clear advantages for studying the pathology of age-related neurological disorders because iPSC-derived iNs reset their epigenetic clocks and therefore may require long-term culturing to mimic aging and re-initiate disease (reviewed in Stricker and Gotz, 2021). Indeed, a direct comparison of iNs derived from iPSCs or tail tip fibroblasts revealed that the latter retained epigenetic signatures of the starting fibroblasts and expressed higher levels of genes associated with DNA damage and oxidative stress compared to iPSC-iNs (Yang et al., 2015). These findings are consistent with the idea that DNA methylation patterns change with biological age (or with cell passages in a dish), establishing a molecular clock that is essentially reset to the beginning after a somatic cell is converted to a pluripotent iPSC state (Horvath, 2013; Bell et al., 2019). In contrast, two studies revealed that the epigenetic signatures of the starting cell are maintained during direct neuronal reprogramming using miR-124 and miR-9/9^∗^ combined with CDM TFs (Huh et al., 2016), or combining the TFs *Neurog2* and *Ascl1* with small molecules (Mertens et al., 2015). Together these studies suggest that direct neuronal reprogramming is better than using iPSC-iNs for modeling diseases, especially those with an aging link.

#### Mitochondrial Age

Mitochondrial function decreases in neurons as they age, and importantly, the reduced OXPHOS capacity is recapitulated in iNs derived from “old” donor fibroblasts that are isolated from individuals 40 years of age and above (Kim et al., 2018). This finding demonstrates that iNs derived through direct neuronal reprogramming, in this case using *Neurog2*, *Ascl1*, and small molecules, can be used to model age related pathologies, including many neurodegenerative diseases (Kim et al., 2018).

#### REST Repressive Complex

Neuronal lineage conversion is less efficient when starting with cells obtained from older vs. younger individuals (Drouin-Ouellet et al., 2017). One study attributed these differences to elevated activity of the REST repressive complex in aged fibroblasts, which prevents the activation of neural-specific mRNAs and miRNAs, including miR-124 and miR-9/9^∗^, highlighted above as critical reprogramming factors (Drouin-Ouellet et al., 2017). The knockdown of REST activity thus offers a path forward for investigators experiencing difficulty in reprogramming fibroblasts from elderly patients.

With these considerations in mind, a snapshot of current efforts to use iNs derived from direct neuronal reprogramming to model neurological disorders is provided in the next section and summarized in Figure 5.

### Disease Modeling Using Patient-Derived iNs

#### Alzheimer’s Disease (AD)

More than 90% of patients with AD exhibit late-onset, sporadic disease with no family history. In comparison, early-onset familial dementia is associated with known mutations in genes such as *PSEN1*, *PSEN2*, and *APP* (reviewed in Nardini et al., 2021). Several groups have now modeled neuronal pathology using iNs derived from sporadic AD patients. The conversion of human fibroblasts to iNs was achieved by expression of the BAM TFs together with *NEUROD1*, with neuronal conversion rates further enhanced by growing cells on nanopatterned substrates (Kim et al., 2017). This conversion strategy was applied either to human fibroblasts with induced expression of a mutant APP or derived from sporadic AD patient fibroblasts with an APOE ε3/4 allele that is an AD risk factor (Kim et al., 2017). Derivative iNs from AD patients exhibited increased amyloid-β42 accumulation and tau hyperphosphorylation, characteristic features of AD, and also displayed an increased vulnerability to peroxide-mediated cell death (Kim et al., 2017). Strikingly, using transcriptomic studies, this study identified DSG2 as an associated risk factor, the knockdown of which could rescue APP processing defects in iNs derived from patient fibroblasts with the APOE ε3/4 allele (Kim et al., 2017).

The generation of iNPCs has also been applied to fibroblasts from patients with sporadic and familial AD, using the six TF and seven TF reprogramming strategies described above (Hou et al., 2017). When the AD-iNPCs were converted to neurons, these neurons also accumulated amyloid-β42 and displayed tau hyperphosphorylation (Hou et al., 2017). Similarly, another group generated iNSCs from fibroblasts isolated from elderly patients with sporadic AD by expressing *SOX2* and culturing the cells in media that contains a variety of small molecules (summarized in Tables 2, 3; Liu and Wang, 2020). The AD patient derived iNSCs could then be differentiated into iNs *in vitro*, and as in the above studies accumulated amyloid-β and high levels of phospho-tau (Liu and Wang, 2020). Future studies could consider the use of these cells for drug screening and additional modeling of disease pathophysiology.

#### Amyotrophic Lateral Sclerosis (ALS)

ALS is a terminal neurodegenerative disease that results in a loss of motor neurons (MNs) in the brain and spinal cord and leads to a fast deterioration of motor function, culminating in death between 3 and 5 years post diagnosis. Upper MNs in layer V of the neocortex degenerate and evidence in animal models and humans support a “dying forward model” of ALS, in which upper MN pathology precedes lower MN loss (Vucic and Kiernan, 2006; Ozdinler et al., 2011; Menon et al., 2015; Thomsen et al., 2018). Most ALS diagnoses are sporadic, with only 5–10% of cases inherited (Pasinelli and Brown, 2006). Of the familial cases, several mutations have been identified in *SOD1*, which encodes for a Cu-Zn superoxide dismutase (Bendotti and Carri, 2004). Hexa-repeat expansion mutations in *chromosome 9 open reading frame 72* (*C9ORF72*) also occurs in ∼50% of familial ALS patients (Choi et al., 2019). Finally, mutations in *TAR DNA binding protein 43* (*TDP-43*) and *fused in sarcoma* (*FUS*) (Lai et al., 2011) are also found in patients with ALS.

To model ALS, iNPCs were generated by misexpressing Yamanaka factors in fibroblasts isolated from patients with a SOD1A4V mutation, C9orf72 expansions, or sporadic disease (Meyer et al., 2013). ALS and control iNPCs were then converted to iAstrocytes, which were co-cultured with motor neurons, which revealed that ALS-derived iAstrocytes, but not glial cells derived from control individuals, induced motor neuron death (Meyer et al., 2013). In a subsequent study, fibroblasts from ALS patients bearing FUS mutations were converted to cholinergic iMNs by overexpressing *NEUROG2*, *SOX11*, *LHX3*, and *ISL1* (Liu et al., 2016). While the ALS iMNs had typical neuronal morphologies and the same marker expression as iMNs derived from healthy individuals, ALS iMNs had an increased propensity to undergo cell death, and displayed electrophysiological deficits (Liu et al., 2016). Finally, as the ALS iMNs could accurately model the pathophysiology of disease, a small molecule screen was also performed, which identified kenpaullone as a factor that increases iMN survival, a promising finding for the future development of new therapeutics (Liu et al., 2016).

#### Huntington Disease (HD)

HD is associated with a poly-glutamine expansion in the Htt protein, which leads to the formation of Htt protein aggregates that are associated with neuronal death (Li and Li, 2004). Expansions of 36 or more CAG repeats, encoding glutamine (Q), in Htt are considered pathogenic (Tabrizi et al., 2020). The first study to apply direct neuronal reprogramming to HD knocked down PTBP1 in fibroblasts from individuals with 16Q (non-pathogenic), 68Q (pathogenic), and 86Q repeats (pathogenic) (Liu et al., 2014). Notably, the iNs derived from individuals that harbor pathogenic Htt polyQ expansions formed Htt aggregates, and displayed abnormally thin and short neurites (Liu et al., 2014). HD patient fibroblast cells that were trans-differentiated to iNPCs using the 6-TF and 7-TF cocktails above were then differentiated into iNs, which displayed pathological hallmarks of disease, including an increase in DNA damage (Hou et al., 2017). Similarly, although not technically direct neuronal reprogramming, an increase in the DNA damage response and higher oxidative stress was observed in iNs derived from HD patient-derived iPSCs (Chiu et al., 2015). Taken together, these studies demonstrate that some features of HD pathophysiology may be accurately modeled by iNs.

#### Parkinson’s Disease (PD)

As with the other neurodegenerative diseases, there are both sporadic and genetic forms of PD (Torrent et al., 2015). The death of dopaminergic neurons in familial PD is associated with oxidative stress and has been linked to several potentially pathogenic mutations in genes involved in mitochondrial homeostasis, such as *PARK2*, *PINK1*, *PARK7*, *SNCA*, *GBA1*, and *LRRK2* (Garcia and Sidransky, 2021). A large number of studies have used iPSC-derived iNs to study PD pathophysiology, with difficulties related to the epigenetic age of these cells (reviewed in Torrent et al., 2015). Direct neuronal reprogramming may circumvent these issues, as highlighted above, and was first applied using the misexpression of Yamanaka factors to generate iNPCs from sporadic and familial PD with a *LRRK2* mutation (Lee et al., 2019). In a follow-up study, PD patient-derived iNPCs displayed a higher rate of apoptosis, which was reduced by treatment with cryptotanshinone (CTN), where CTN modulates mitochondrial ROS and membrane potential to reduce cellular apoptosis (Lee J. E. et al., 2020). Follow-up studies are required to determine whether iNs derived from the PD patient iNPCs display pathological features.

#### Spinal Muscular Atrophy (SMA)

SMA is an autosomal recessive disorder associated with a homozygous mutation of *SMN1*, and results in spinal motor neuron death. The pathophysiology of SMA has recently been characterized by generating iMNs from patient and control fibroblasts through the expression of a cocktail of eight TFs: *ASCL1*, *ISL1*, *NEUROD1*, *BRN2*, *HB9*, *LHX3*, *MYT1L*, and *NEUROG2* (Zhang et al., 2017). Notably, SMA patient-derived iMNs had a reduced rate of neurite outgrowth, and displayed signs of neurite degeneration in long-term culture. These features highlight the potential of this model to further understand disease biology (Zhang et al., 2017).

#### Niemann-Pick Disease Type C (NPDC)

NPDC is an autosomal recessive neurodegenerative disease associated with homozygous mutations in *NPC1* or *NPC2* (Carstea et al., 1997). NPDC is a liposomal storage disorder, and manifests early in life with dementia, cerebellar ataxia and other symptoms that progresses ultimately to death. Modeling NPDC has been achieved using the direct conversion of patient fibroblasts to iNPCs by misexpressing *SOX2* and the high mobility group A2 (*HMGA2*) protein, which alters chromatin structure by binding to AT rich regions of the genome (Sung et al., 2017). Prior studies have demonstrated that *HMGA2* augments *SOX2’s* ability to reprogram somatic cells to iNSCs (Chang et al., 2005). In this study, iNPCs derived from NPDC patients were shown to have defects in self-renewal, neurogenesis, and cholesterol homeostasis (Sung et al., 2017). As the patient-derived iNSCs phenocopy several aspects of NPDC, future studies can examine their utility for drug screening.

#### Neuropsychiatric Disorders

Neuropsychiatric disorders are diverse in origin and in manifestation, and lead to a variety of defects, such as in cognition, psychoses, neuroses, and mental impairment. In many cases, the etiology of these disorders remains unknown or poorly characterized. A large number of studies have linked miRNA disruptions to schizophrenia, autism spectrum disorder, depression, and other neuropsychiatric disorders (reviewed in Bavamian et al., 2015). One example is bipolar disorder (BD), in which patient may exhibit cycles of mania followed by depressive episodes. The generation of iNs from BD patients and healthy controls was achieved by misexpressing miR-9, miR-124, *NEUROD2*, *ASCL1*, and *MYT1L* (Bavamian et al., 2015). The iNs from BD patients expressed elevated levels of miR-34a, which was also increased in autopsy specimens (Bavamian et al., 2015). Consequently, miR-34a target genes, such as *ANK3* and *CACNB3*, were elevated in BD patient-derived iNs. This study highlights the potential of utilizing neuronal reprogramming to identify the genetic contributors of a neuropsychiatric disorder.

## *In vivo* Neuronal Reprogramming of Endogenous Glial Cells

### Predominant Use of bHLH Transcription Factors for *in vivo* Reprogramming

**FIGURE 6:**
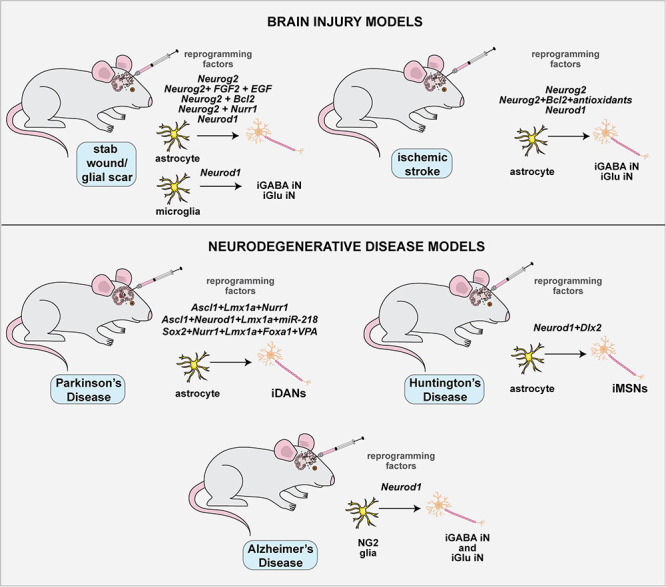
Applications of *in vivo* direct neuronal reprogramming to treat injury and/or neurodegenerative disease. Direct neuronal reprogramming has therapeutic potential for brain injury (e.g., glial scars, stroke) and neurodegenerative diseases, such as PD, HD, and ALS. The advantage of this approach is that endogenous glial cells can be directly reprogrammed to replace lost neurons at the site of injury or trauma.

The first evidence that endogenous glial cells could be converted to iNs by TF misexpression was obtained in a stab wound injury model in the adult mouse brain, with *Pax6* driving the neuronal conversion of resident glial cells (Buffo et al., 2005). Since then, *in vivo* reprogramming of reactive glia, including NG2 glia and astrocytes, to iNs was achieved by expressing a variety of TFs alone or in combination with other molecules, but the factors used most often are bHLH TFs. Critical studies on these bHLH TFs are summarized herein and in Figure 6.

#### Employing *Neurog2* for *in vivo* Neuronal Reprogramming

*Neurog2* alone is inherently inefficient at inducing neuronal lineage conversion, especially *in vivo*, but several groups have enhanced reprogramming by combining *Neurog2* with various other factors. Specifically, *in vivo* neuronal reprogramming has been enhanced by expressing *Neurog2* in glial cells together with the co-injection of growth factors (FGF2, EGF) (Grande et al., 2013), co-injection of DAPT to block Notch signaling (Hu et al., 2019), co-expression with *Bcl2* and injection of antioxidants (Gascon et al., 2016), or through co-expression with *Nurr1* (Mattugini et al., 2019). Notably, a study of focal ischemia, a well-characterized stroke model, further enhanced the ability of *Neurog2* to drive glial conversion to mature DCX-/NEUN+ iNs compared to the immature DCX+ iNs that predominated in the uninjured brain (Grande et al., 2013). In contrast, in a conflicting report, the misexpression of *Neurog2* and *Bcl2* in reactive astrocytes post ischemia led to very low neuronal conversion rates, both in young and old mice (Gresita et al., 2019).

These studies suggest that the astrocyte changes that accompany injury, such as reactive gliosis, may impact glial cell plasticity and hence influence neuronal reprogramming. Moreover, astrocytes in different brain regions have a differential capacity to undergo neuronal lineage conversion, with *Neurog2* triggering the conversion of GFAP+ glial cells to iNs more effectively in the cortex > cerebellum > spinal cord (Hu et al., 2019). These studies highlight the sensitivity of reprogramming TFs to environmental context, and suggest that their activities are tightly regulated. Such a tight regulation fits with the regulatory controls that govern neural-specific bHLH function during normal embryonic and adult neurogenesis (reviewed in Oproescu et al., 2021).

#### Use of *Ascl1* for *in vivo* Neuronal Reprogramming

In a 2014 study, resident NG2 glia were induced to differentiate into neurons either by the overexpression of *Ascl1* with *Sox2*, or *Sox2* alone, triggering the generation of both immature DCX+ or mature DCX-/NEUN+ cells that were electrically active based on patch clamp recordings (Heinrich et al., 2014). *Ascl1* has also been combined with *Lmx1a* and *Nurr1* (ALN) to convert endogenous NG2 glia to inhibitory iNs in a dopamine-depleted (6-OHDA treated) mouse model of Parkinson’s disease (Pereira et al., 2017). The ALN-iNs integrated into the existing neuronal circuitry in the striatum and midbrain of 6-OHDA treated mice (Pereira et al., 2017). Interestingly, although this same combination of factors generated dopaminergic iNs *in vitro* (Torper et al., 2013), it could not activate tyrosine hydroxylase (TH) expression in iNs generated *in vivo* (Pereira et al., 2017). In contrast, the combination of a slightly different combination of TFs (*Ascl1*, *NeuroD1*, *Lmx1a*) and miR-218 efficiently programmed striatal astrocytes into functional induced dopaminergic neurons (iDANs), also in a 6-OHDA model of Parkinson’s disease (Rivetti di Val Cervo et al., 2017). Not only were the iDANs able to fire action potentials, but there was also an associated behavioral recovery based on improved performance on motor tasks (Rivetti di Val Cervo et al., 2017). However, one confounding factor is that this study did not use a genetic reporter for the reprogrammed iNs, so some concern has been expressed that the identified TH+ cells arose *in situ* from the 6-OHDA lesion itself (Pereira et al., 2017).

Nevertheless, these studies hold promise that a neurodegenerative disease such as Parkinson’s disease may be treated by direct neuronal reprogramming in the future. Indeed, improvements have been made in the TF cocktails used for iDAN reprogramming. For example, by misexpressing *Sox2*, *Nurr1*, *Lmx1a*, and *Foxa1* TFs and injecting Valproic acid, striatal glia could be converted to iDANs *in vivo* (Niu et al., 2018). It thus seems only a matter of time until an effective TF combination is found to generate iDANs *in vivo* for the treatment of PD.

#### Use of *Neurod1* for *in vivo* Neuronal Reprogramming

*Neurod1* has proven to be a potent reprogramming vehicle *in vivo*, driving the conversion of activated astrocytes or NG2 glia into functional iNs in a stab wound brain injury model and in Alzheimer’s disease model mice (Guo Z. et al., 2014). While *Neurod1* effectively converts cortical astrocytes to glutamatergic iNs, consistent with its subtype differentiation properties in development, *Neurod1* converted NG2 glia to glutamatergic and GABAergic iNs (Guo Z. et al., 2014). Notably, *Neurod1* could also convert striatal microglia to functional neurons *in vivo*, even without intended injury, although injecting the viral construct may itself trigger a stab wound response (Matsuda et al., 2019). To avoid this confounding factor, *Neurod1* was delivered intravascularly to the brain by packaging in adeno-associated virus (AAV) 9, which crosses the blood-brain-barrier without the need for injections into the brain. With this mode of delivery, non-reactive astrocytes in the striatum were reprogrammed to iNs (Brulet et al., 2017). Surprisingly, however, cortical astrocytes were not converted to iNs with the intravascular AAV9 delivery approach (Brulet et al., 2017). These findings are consistent with the demonstration that astrocytes show region-specific responses to neuronal reprogramming factors (Hu et al., 2019).

Glial scars form after injury to form a functional barrier between the injured and healthy brain. While they serve an important role in the injury response, they also permanently remodel the brain and can be detrimental in the long-term (Sofroniew, 2015). Interestingly, the overexpression of *Neurod1* in astrocytes generated functional iNs and reduced glial toxicity after a stab wound brain injury (Zhang et al., 2020). Moreover, the use of the GFAP promoter in this study appeared to more selectively target the conversion of A1 astrocytes, which secrete inflammatory cytokines, vs. the A2 astrocytes, which are neuroprotective (Zhang et al., 2020). There was also significant tissue repair in the wounded region of the brain after *Neurod1* expression, including rebalancing of the neuron: astrocyte ratio, improvement of the blood-brain-barrier, and a decrease in both reactive astrocytes and microglia (Zhang et al., 2020).

*Neurod1*-induced lineage conversion of reactive astrocytes has also been applied to the treatment of a focal stroke model in mice induced using endothelin-1, a vasoconstrictor (Chen et al., 2020; Livingston et al., 2020). In both of these studies, *Neurod1*-induced the effective transdifferentiation of astrocytes to functional iNs *in vivo*, and provided a significant enhancement of motor behavior (Chen et al., 2020; Livingston et al., 2020). These studies provide important proof-of-concept evidence that a neuronal reprogramming strategy could be applied to the treatment of brain injury.

Finally, *Neurod1*-induced neuronal reprogramming has also been applied to the treatment of a neurodegenerative disease. When *Neurod1* was co-expressed with *Dlx2*, it could drive the efficient conversion of striatal astrocytes to GABAergic, induced medium spiny neurons (iMSNs) in a mouse model of Huntington Disease (R6/2 mice) (Wu et al., 2020). Not only were the iMSNs functional, but this gene therapy also extended the lifespan of R6/2 mice, delayed their onset of motor deficits, and caused reduced neuronal pathology, which provides encouraging support for the potential of neuronal reprogramming to treat neurodegeneration (Wu et al., 2020).

### Therapeutic Potential of *in vivo* Reprogramming in Comparison to the Current Strategies

#### Comparing the Potential of Exogenous vs. Endogenous Neuronal Repair

In neurodegenerative diseases, dying neurons are not replaced, so new therapeutic options are required. Similarly, even though some neurogenesis occurs in the infarct region post-stroke, due to NSC migration from neurogenic zones in the SGZ and V-SVZ, very few new neurons are made and functional recovery is not complete (Rahman et al., 2021). New strategies that drive neuronal replacement are thus required. When devising neuronal replacement strategies for brain injury or neurodegenerative diseases, there are two potential approaches—endogenous or exogenous repair. In this review, we focus on the potential of direct neuronal reprogramming, which could be used to target endogenous glial cells, or to generate iNs for transplant and exogenous repair.

##### Potential of Exogenous Repair

Cell transplantation in the CNS has long been considered as a potential therapeutic approach, and could in theory be applied to iNs. Indeed, stem cell transplantations into the brain have been investigated since the 1980s, with several human clinical trials initiated (reviewed in Fan et al., 2020; Osborn et al., 2020; Stoddard-Bennett and Pera, 2020). For instance, a search of https://clinicaltrials.gov/ for “stem cell” and various neurological disorders revealed that 31 clinical trials have been initiated for PD, 60 for ALS, and 93 for stroke. Yet, despite these many trials, stem cell transplants are not yet standard-of-care treatments for any of these disorders. Many noteworthy challenges exist, including cell source and purity, potential tumorigenicity, and immune tolerance (Michelsen et al., 2015; Doerr et al., 2017). Cell purity is of particular concern when transplanting neurons derived from pluripotent cells, which are potentially tumorigenic, so the development of robust strategies to remove undifferentiated cells is critical. Interestingly, by linking a cell death gene (*Tk, thymidine kinase*) to the promoter of a cell division gene (*Cdk1*), one group has devised a strategy that can kill any proliferating cells that may have escaped cell purification steps (Liang Q. et al., 2018).

While immune rejection could be prevented by using autologous cell transplantations, for instance of iPSC-derived cells (reviewed in Osborn et al., 2020), the hope would be that a universal supply of iNs for transplant could be developed. To avoid lifelong immunosuppression, one approach would be to generate a panel of human leukocyte antigen (HLA)-typed iPSCs that could be matched to patients (Doi et al., 2020; Piao et al., 2021). Another innovative approach to tackle the issue of host-immunoreactivity and graft rejection has involved the development of a “cell cloaking” strategy, which involves the expression of eight immunomodulatory genes, allowing transplanted cells to escape rejection in allogeneic hosts (Harding et al., 2019; Lanza et al., 2019). Future extensive studies will be required to develop these approaches for clinical application.

##### Potential of Endogenous Repair

The main advantage of neuronal reprogramming *in situ* is the possibility that resident glial cells in the brain can be targeted, so that brain rejection is not an issue. The ability to move forward with an endogenous repair approach is supported by major advances made in AAV gene therapy, including many examples now of AAVs moving to the clinic, especially to treat monogenic diseases. Some examples for CNS disorders include an AAV2 gene therapy designed to express RPE65 in the eye to treat Leber Congenital Amaurosis (Luxturna^®^) and an AAV9 vector that expresses SMN1 in motor neurons to treat Spinal Muscular Atrophy (Spinaraza^®^).

#### Future Challenges and Safety Considerations for Endogenous Neuronal Repair

##### Effects of Astrocyte Loss/Neuronal Gain

The main approach for neuronal reprogramming is to convert astrocytes to iNs, but an important consideration is the effect of the consequent astrocyte loss on brain function. Indeed, there is some evidence that the loss of local astrocytes affects brain homeostasis in PD patients (reviewed in Khakh and Deneen, 2019). Future studies will be required to investigate the consequences of glial loss in detail. It is also important to consider that iNs may form aberrant connections that are counterproductive, or even detrimental, triggering epileptic foci, for example.

##### Toxicity of Overexpressed TFs

An early study used retroviruses to overexpress several neural bHLH TFs, including *Neurog2*, *Ascl1*, and *Neurod1*, in the P0/P1 cortical subventricular zone, and while these genes efficiently induced neurogenesis, the newly derived neurons ultimately underwent apoptosis (Cai et al., 2000). This study suggests that the long-term expression of developmental bHLH genes in mature neurons is ultimately toxic. It is therefore important to devise reprogramming strategies that prevent sustained expression of bHLH genes. While most studies use a GFAP astrocytic promoter to drive bHLH gene expression, which theoretically should not be active in the new iNs, detailed studies are required to test this assumption.

##### Cellular Context

An important consideration for moving neuronal reprogramming into the clinic is that the TFs and miRNAs that trigger neuronal lineage conversion all function in a context-dependent fashion. Understanding how the diseased brain environment affects their functions is thus critical. For instance, *Ascl1* is a potent neuronal reprogramming factor in fibroblasts (Vierbuchen et al., 2010; Caiazzo et al., 2011; Kim et al., 2011b; Pang et al., 2011; Pfisterer et al., 2011a; Son et al., 2011), hepatocytes (Marro et al., 2011), cardiomyocytes (Chuang et al., 2017), astrocytes (Rivetti di Val Cervo et al., 2017), and pluripotent cells (Yang et al., 2017), but not in the adult neocortex (Grande et al., 2013), hippocampus and spinal cord (Ohori et al., 2006; Jessberger et al., 2008). Similarly, *Neurog2* is used less often for neuronal reprogramming because it must be combined with other signals to become a potent lineage converter (Gascon et al., 2016). It currently remains to be determined how the diseased brain affects neuronal reprogramming efficiency. When considering how TFs/miRNAs operate in a diseased brain, especially *in vivo*, one must consider that cell fate is ultimately dictated by the balance of inducers and repressors (Yamashita et al., 2019). The impact of several negative regulators of neurogenesis (e.g., p53-p21 pathway, REST repressor complex, oxidative stress) that may suppress reprogramming processes in a diseased brain remain to be fully elucidated (Cheloufi et al., 2015; Jiang et al., 2015; Masserdotti et al., 2015; Gascon et al., 2016). Addressing the “real life” hurdles of the compromised *in vivo* environment of the diseased/damaged brain will be essential to successfully using neuronal reprogramming for neural repair in the clinic.

##### Long-Term Toxicity of AAVs

AAVs delivered to the brain may trigger an immune reaction *in situ*, or if the blood-brain-barrier is compromised, which generally occurs in neurological diseases, escape into the CSF, blood or draining lymph nodes to infect peripheral organs. Potential AAV toxicity must, therefore be tested extensively before moving to the clinic.

##### Delivery Challenges

Our ability to deliver therapeutics to the brain is limited by the blood-brain-barrier (BBB), which blocks all but the smallest molecules from entering *via* the bloodstream. Intracranial injections are thus currently the method of choice for viral delivery to the brain in animal models, and while this approach can be used in humans, it is surgically invasive. Magnetic resonance imaging (MRI)-guided focused ultrasound (FUS) is a new, minimally invasive approach that transiently increases the permeability of the BBB to provide therapeutic access to the brain. FUS has been used for delivery of antibodies (Jordao et al., 2010), genes (Thevenot et al., 2012; Weber-Adrian et al., 2015), stem cells (Burgess et al., 2011), and immunoglobulins (Dubey et al., 2020) to the brain and spinal cord in mouse models. Moreover, FUS has been used to transiently permeabilize the BBB in human patients with ALS (Abrahao et al., 2019) or AD (Lipsman et al., 2018; Meng et al., 2019). Future investigations using FUS-mediated AAV gene delivery, or other minimally invasive techniques, will improve the feasibility of using gene therapies for direct neuronal reprogramming.

## Conclusion

The development of experimental paradigms for direct somatic cell reprogramming into any desired lineage, without a transient pluripotent state, was a major breakthrough in the field of lineage conversion. Remarkable achievements have been made in identifying TFs, miRNAs, small molecules and other factors that drive direct neuronal reprogramming, both *in vitro* and *in vivo.* The potential application of this technology to therapeutic scenarios is just coming to the forefront, and provides new promise for the large number of individuals afflicted by neurodegenerative disease or brain injury.

## Author Contributions

LV and CS: conceptualization. LV, EP, LD, and CS: writing—review and editing. TF: artwork. CS: funding acquisition. All authors contributed to the article and approved the submitted version.

## Conflict of Interest

The authors declare that the research was conducted in the absence of any commercial or financial relationships that could be construed as a potential conflict of interest.

## Abbreviations

AD: Alzheimer’s disease
ALS: Amyotrophic lateral sclerosis
ANL: *Ascl1-Nurr1-Lmx1a*
BAF: Brg/Brm-associated factors
BAM: Brn2-Ascl1-Myt1l
BDNF: Brain derived neurotrophic factor
bHLH: basic-helix-loop-helix
BMP: Bone morphogenic protein
CNS: central nervous system
CREB: cyclic-AMP response element binding protein
CTN: Cryptotanshinone
DALY: disability-adjusted life years
DNMT: DNA methyltransferase
DREADD: Designer Receptors Exclusively Activated by Designer Drugs
ESC: embryonic stem cells
FGF: fibroblast growth factor
FTD: frontotemporal dementia
GDNF: Glial cell derived neurotrophic factor
GFAP: glial fibrillary acidic protein
HAFs: human adult fibroblasts
HD: Huntington’s disease
HDAC: histone deacetylase
hESC: human embryonic stem cell
iDA: induced dopaminergic neurons
iMNs: induced motor neurons
iN: induced neurons
iPSC: induced pluripotent stem cell
JHDM: Jumonji-C domain histone demethylases
LC-MS: liquid chromatography- mass spectroscopy
LIF: Leukemia inhibitory factor
lncRNA: long non-coding RNA
MCAO: middle cerebral artery occlusion
MEF: mouse embryonic fibroblasts
miR: microRNA
*Mll1*: *Mixed lineage leukemia-1*
MRIgFUS: magnetic resonance guided focused ultrasound
MSN: medium spiny neurons
NaB: sodium butyrate
NAMPT: nicotinamide phosphoribosyl transferase*Neurog2*, *Neurogenin 2*
NICD: Notch intracellular domain
NPC: Neural progenitor cell
NPDC: Niemann-Pick disease type C
NSC: Neural stem cell
OEC: Olfactory Ensheathing cells
OHDA: hydroxydopamine
OPC: oligodendrocyte precursor cells
OXPHOS: oxidative phosphorylation
P: postnatal
PD: Parkinson’s disease
PNS: peripheral nervous system
PTBP: poly-pyrimidine-tract-binding protein
RA: Retinoic acid
REST: (RE1) silencing transcription factor
ROS: reactive oxygen species
SGZ: subgranular zone
SHH: sonic hedgehog
SMA: spinal muscular atrophy
TET: ten-eleven translocation
Tet3: TET methylcytosine dioxygenase3
TF: transcription factor
TGFb: Transforming growth factor b
TIGAR: TP53 inducible glycolysis and apoptosis regulator
UMSC: Human umbilical cord mesenchymal stem cells
VPA: valproic acid
V-SVZ: ventricular-subventricular zone
YAP: Yes-associated protein.
